# Designing next generation recombinant protein expression platforms by modulating the cellular stress response in *Escherichia coli*

**DOI:** 10.1186/s12934-020-01488-w

**Published:** 2020-12-11

**Authors:** Richa Guleria, Priyanka Jain, Madhulika Verma, Krishna J. Mukherjee

**Affiliations:** 1 School of Biotechnology, Jawaharlal Nehru University, New Delhi, 110067 India; 2 School of Computational and Integrative Sciences, Jawaharlal Nehru University, New Delhi, 110067 India; 3 Department of Biochemical Engineering and Biotechnology, Indian Institute of Technology Delhi, New Delhi, 110016 India

**Keywords:** Cellular stress response, *Escherichia coli*, Knockouts, Recombinant protein expression, Signaling, Transcriptome

## Abstract

**Background:**

A cellular stress response (CSR) is triggered upon recombinant protein synthesis which acts as a global feedback regulator of protein expression. To remove this key regulatory bottleneck, we had previously proposed that genes that are up-regulated post induction could be part of the signaling pathways which activate the CSR. Knocking out some of these genes which were non-essential and belonged to the bottom of the *E. coli* regulatory network had provided higher expression of GFP and *L-asparaginase*.

**Results:**

We chose the best performing double knockout *E. coli* BW25113*ΔelaAΔcysW* and demonstrated its ability to enhance the expression of the toxic Rubella E1 glycoprotein by 2.5-fold by tagging it with *sf*GFP at the C-terminal end to better quantify expression levels. Transcriptomic analysis of this hyper-expressing mutant showed that a significantly lower proportion of genes got down-regulated post induction, which included genes for transcription, translation, protein folding and sorting, ribosome biogenesis, carbon metabolism, amino acid and ATP synthesis. This down-regulation which is a typical feature of the CSR was clearly blocked in the double knockout strain leading to its enhanced expression capability. Finally, we supplemented the expression of substrate uptake genes *glpK* and *glpD* whose down-regulation was not prevented in the double knockout, thus ameliorating almost all the negative effects of the CSR and obtained a further doubling in recombinant protein yields.

**Conclusion:**

The study validated the hypothesis that these up-regulated genes act as signaling messengers which activate the CSR and thus, despite having no casual connection with recombinant protein synthesis, can improve cellular health and protein expression capabilities. Combining gene knockouts with supplementing the expression of key down-regulated genes can counter the harmful effects of CSR and help in the design of a truly superior host platform for recombinant protein expression.

## Background

Protein synthesis is an energy intensive process and the diversion of metabolites and energy for recombinant protein production elicits a cellular stress response (CSR) [1, 2], which combines the features of the generalized stress response, the heat shock, oxidative stress and the stringent response [3–6]. This CSR can be perceived as a defense mechanism by which the cell safeguards itself from allocating too many resources to a single process which can be detrimental to its survival [7].

The detailed mechanism of how exactly the cell senses this stress and takes corrective action is yet to be deciphered. We do not therefore know how to intervene and modulate this stress response so as to ensure that the metabolic and energy flux required for protein synthesis continues to remain available. That is why usually the highest rates of recombinant protein synthesis are observed only for 2–4 h post induction after which it declines sharply along with a concomitant drop in growth rates [8–11]. A much better picture of cellular dynamics has emerged by transcriptomic profiling of post induction cultures [4, 9, 12–14]. These have been combined with proteomic and metabolomics studies to show that increased acetate production, growth retardation, increased demand for maintenance energy, the down-regulation of amino acid biosynthesis, poorer substrate uptake and change in the pattern of oxygen utilization are all effects of cellular reprogramming due to stress [15–19]. This reprogramming works to reduce the rate of protein synthesis and hence many researchers have supplemented the expression of key down-regulated genes such as those involved in ATP synthesis, energy generation, substrate uptake and obtained significant increase in productivity [18, 20–22]. However, the main disadvantage of this strategy is that a very large number of genes get down-regulated leading to an almost complete shutdown of cellular activity and it is a near impossible task to simultaneously supplement the activity of so many genes.

Till now we have not made the more ambitious attempt of trying to block the initiation of the CSR, which could ideally preclude all these undesirable effects. In a previous study, we tried to achieve this by identifying up-regulated genes as potential candidates which signal the onset of the CSR [23]. It is important to note that in order to prevent cascading effects we had selected genes with no known downstream regulates and no direct relationship with the protein synthesis process. We showed that some of these knockouts improved protein expression and combining these to create double knockouts (DKOs) helped in further enhancing protein yields. However, many questions remained unanswered. First, since we had only tested two recombinant proteins; GFP and *L-asparaginase*, we were unsure whether the beneficial effects of these knockouts would extend to the improved expression of a wider range of proteins especially “difficult to express” proteins whose commercialization potential often remains unrealized due to poor yields. Second, we had no direct proof that the increase in expression was brought about by the blocking of the CSR. This could only be established by comparative transcriptomic profiling if we could demonstrate that those genes which are known to get down-regulated due to the onset of the CSR remain relatively unchanged in the knockout strain. These should include critically important genes which have a directly impact on protein synthesis. However, as is well known, there are multiple signaling pathways that are triggered during a stress response [4, 24–27] and knocking out a couple of genes, even if they were part of the signaling pathway, would not abolish all such pathways. We thus expected only a partial triggering of the CSR leading to a significantly smaller subset of genes getting down-regulated in the knockout strain. Interestingly this was precisely what was observed and we found that only a few critical genes got down-regulated, important among them those belonging to substrate uptake. We could therefore simply supplement the expression of these genes thereby ensuring that almost all the negative effects of the CSR got resolved. This complementation further increased the expression levels of the target protein *L-asparaginase* and confirmed that this gene knockout strategy combined with gene supplementation has the potential to help in the design of next generation platforms for recombinant protein expression.

## Results and discussion

In a previous study, we had hypothesized that some of the non-essential genes, which get up-regulated in post induction cultures, may be acting as signaling messengers which activate the CSR. Knocking them out would disrupt this signaling pathway leading to a lowered CSR and hence higher protein yields. In a preliminary proof of principle study, we generated a panel of single and double gene knockouts that gave superior expression for GFP and *L-asparaginase* [23]. To validate this study and demonstrate that these knock outs indeed constituted a better expression platform, we decided to check their ability to express a “difficult to express” protein. For this task we chose a DKO combination ‘*ΔelaAΔcysW’* which had shown improved performance and tested its ability to enhance the expression of ‘Rubella E1 glycoprotein’ which is otherwise expressed very poorly due to its toxic nature.

### Expression studies of Rubella E1-*sfGFP*

**Fig. 1:**
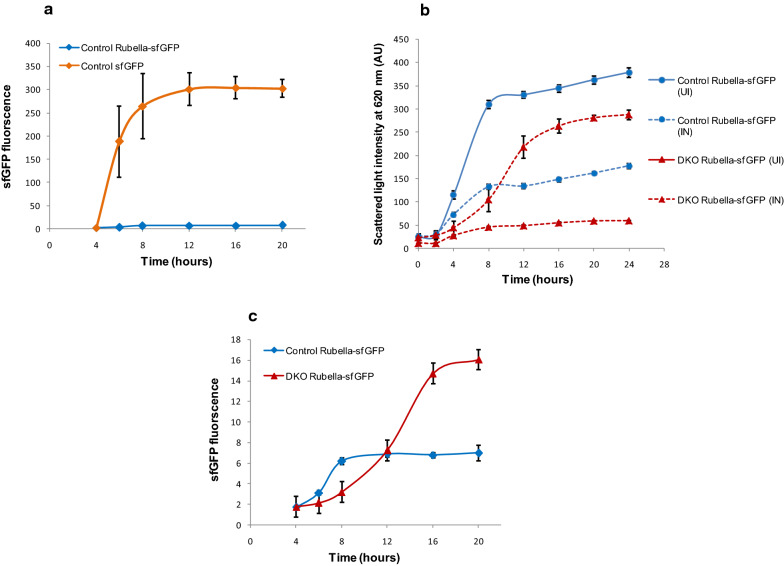
GFP fluorescence, growth and product profile in control and double knockout (DKO) strains. Control and DKO strains were transformed with plasmids carrying genes for *sfGFP* and *RubellaE1*-*sfGFP,* and grown in TB media supplemented with 0.4% v/v glycerol and 10 mM MgSO_4_. (**a**) GFP fluorescence profile (Excitation λ: 485 nm, Emission λ: 507 nm) in AU (arbitrary units) for control strain expressing *sfGFP* (orange) and *Rubella E1-sfGFP* (blue). **b** Growth profiles (biomass concentration measured in arbitrary units (AUs) by scattered light intensity at 620 nm) of control (blue) and DKO strain (red) containing *pBAD-Rubella E1-sfGFP* expression vector under induced (dotted lines) and uninduced (solid lines) conditions. **c** GFP fluorescence levels in control (blue) and DKO (red) strains expressing the Rubella *sfGFP* fusion protein. Data represents mean ± SD of three independent experiments

The Rubella E1 gene was initially cloned downstream of the “*araBAD*” promoter in a *pBAD24* vector and expression was checked in shake flask cultures. However, extremely low levels of expression were obtained and the heterologous protein could only be located in post induction cultures by Western blot experiments using Rubella E1 antibody (data not shown). Since the quantitation of differential levels of expression using relative intensities of Western blot bands is known to be problematic, we decided instead to tag this protein at the C-terminal end with *sfGFP* and measure relative fluorescence levels. The expression level of this fusion protein was 40-fold lower compared to *sfGFP* alone (Fig. 1a), clearly demonstrating that the fusion protein retained the characteristic toxic nature of the original Rubella E1 protein. To compare expression levels, the plasmid containing the ‘Rubella E1-*sfGFP’* gene under the *pBAD* promoter was transformed into both control & DKO strains and the expression levels were checked online by measuring *sfGFP* fluorescence in a microbioreactor. We observed a significant decline in growth rate post-induction for both strains, an indication of the toxic effect of this fusion protein on cellular heath (Fig. 1b).

The maximum GFP expression obtained in the DKO was 16 AU (arbitrary units) which was about 2.5-fold higher compared to the control. Interestingly, the DKO showed a continuous increase in fluorescence for a significantly longer time, i.e. 16 h, in comparison to control where the *sfGFP* fluorescence plateaued within 8 h post induction (Fig. 1c). These results suggest that the DKO was able to counter the stress associated with toxic protein expression, leading to a 5.6-fold increase in product accumulation per unit biomass. Simultaneously the ability to sustain expression for longer periods, that we had observed with *L-asparaginase* as well, indicated that the global feedback controls which regulate protein expression are weaker in this DKO strain.

### Transcriptomic studies

We conducted a comparative transcriptomic study of pre and post induction cultures expressing *L-asparaginase* in the DKO and control strains to check the impact of these gene knockouts on the CSR. We used complex rather than defined media since it provides a much higher levels of recombinant protein expression and therefore possibly triggers a stronger CSR. Our hypothesis was that this CSR would be partially blocked in the DKO strain and hence a significantly lower proportion of genes would show differential expression post induction.

### Analysis of down-regulated genes in the DKO and control

**Fig. 2:**
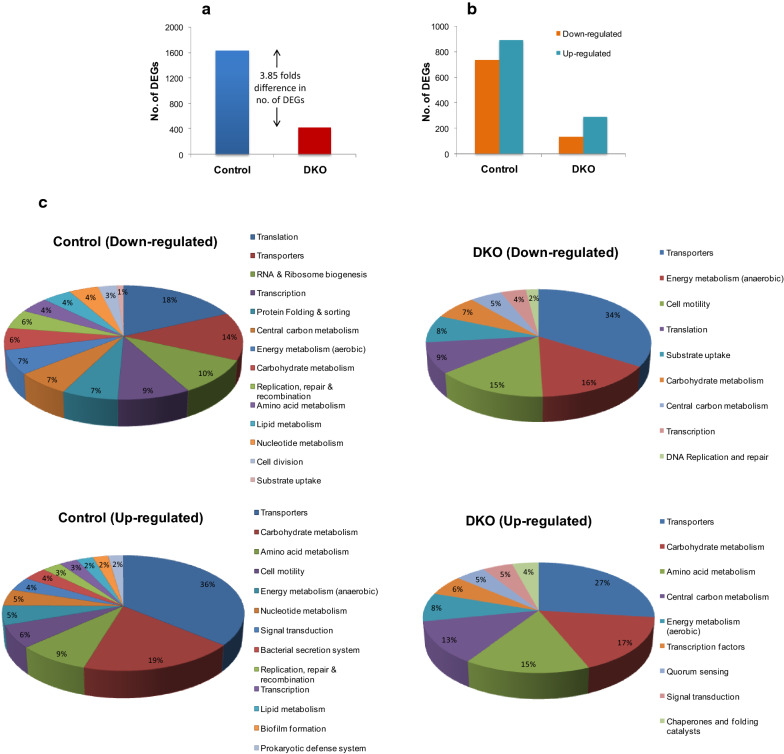
Categorization of DEGs pre and post induction for control and DKO obtained by transcriptomic analysis. **a** Plot of total no. of DEGs obtained for control (blue) and DKO (red) using a cut-off criterion of |log_2_(X_IN_/X_UN_)|> 1. **b** Plot showing number of up-regulated and down-regulated genes separately in control and DKO strain. **c** Functional categorization of up and down-regulated gene clusters for control and DKO strains using KEGG database

To obtain a global picture of the changes post induction in the DKO and control strains we applied a cutoff of |log_2_(X_IN_/X_UN_)|> 1 i.e. a fold change magnitude of ≥ 2 in terms of either up or down-regulation, to obtain the differentially expressed genes (DEGs). We found only 423 DEGs in the DKO strain in contrast to 1632 DEGs in control (Fig. 2a). This was a truly remarkable result given that knocking out just two genes that too belonging to the bottom of the regulatory hierarchy of *E. coli,* could lead to such a large difference in the number of differentially regulated genes. It was also the first clear proof that the reprogramming of the cellular machinery which is the primary effect of the CSR was significantly reduced in the DKO strain.

**Fig. 3:**
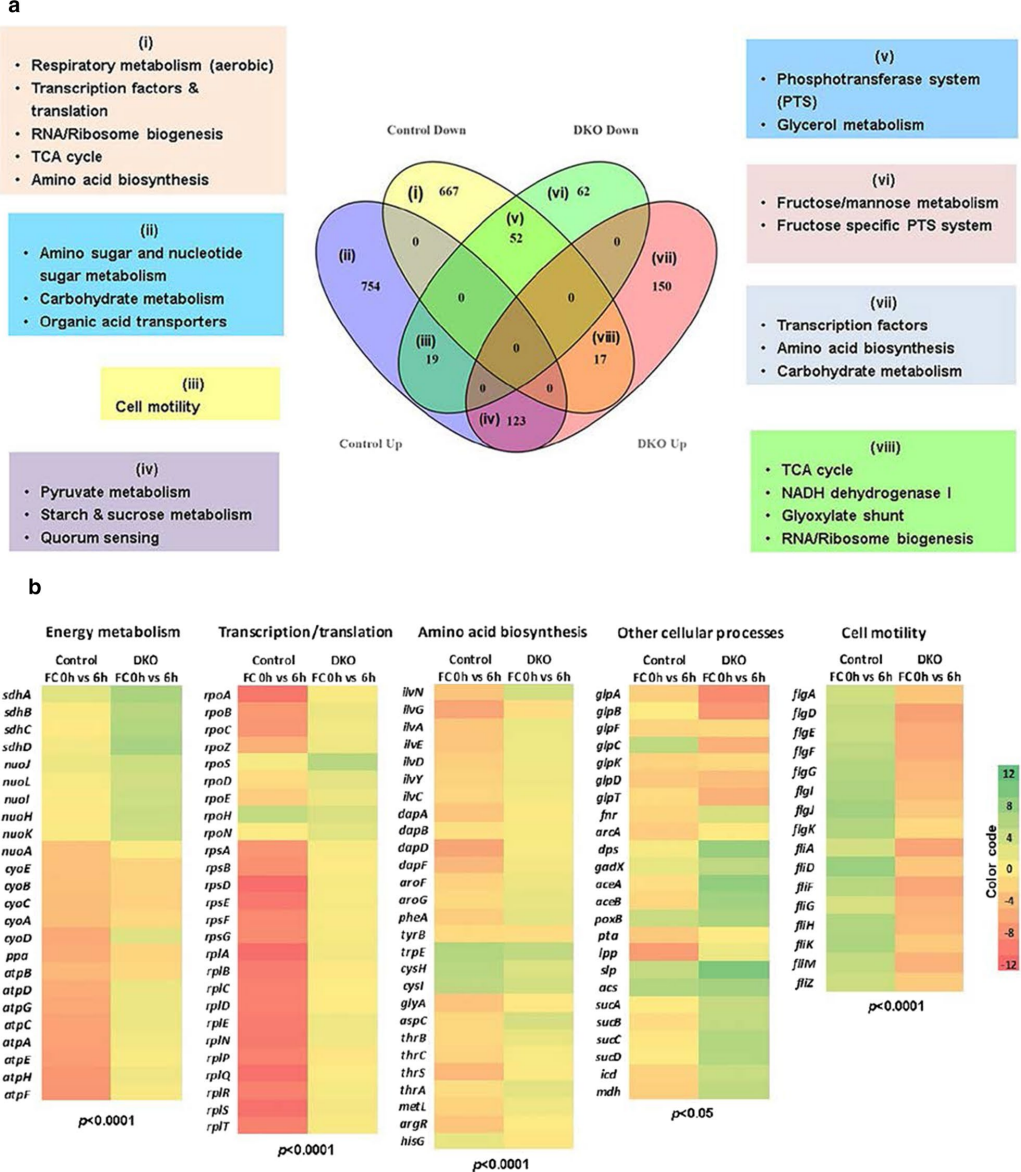
Relationship between DEGs obtained for control and DKO strains. **a** Venn diagram of common and unique DEGs representing: (i) gene set down-regulated only in control (ii) gene set up-regulated only in control; (iii) gene set up-regulated in control but down-regulated in DKO; (iv) common gene set up-regulated in both control and DKO; (v) common gene set down-regulated in both control and DKO; (vi) gene set down-regulated only in DKO; (vii) gene set up-regulated only in DKO; and (viii) gene set down-regulated in control but up-regulated in DKO. **b** Heat maps showing fold change in expression of selected genes in control and DKO belonging to following major categories: energy metabolism, transcription & translation, amino acid biosynthesis, other cellular processes (includes substrate uptake, stress resistance and TCA cycle genes) and cell motility. FC represents “fold change”. The results in ‘b’ were analyzed using a paired *t-test* (***P < 0.0001, ***P < 0.0001, ***P < 0.0001, *P < 0.05, ***P < 0.0001)

However, before arriving at any firm conclusions it was important to analyze the nature of these DEGs. From the 1632 DEGs in control, 736 were found to be down-regulated (Fig. 2b); out of which 667 genes were specific to the control. These down-regulated and up-regulated gene clusters were functionally categorized using KEGG GENES database. The majority of down-regulated genes were associated with key cellular processes like translation (90 DEGs), transcription (44 DEGs), RNA and ribosome biogenesis (50 DEGs), transport (67 DEGs), protein folding, sorting and degradation processes (34 DEGs), central carbon metabolism (33 DEGs), energy metabolism (34 DEGs), DNA replication, repair and recombination (27 DEGs), glycerol (substrate) metabolism (5 DEGs) and other catabolic processes (97 DEGs; carbohydrate, amino acid and nucleotide metabolism) (Fig. 2c). This pattern was similar to what we had observed in our previous transcriptomic studies conducted on high cell density fed batch cultures expressing *L-asparaginase* and other recombinant proteins [4, 14, 28]. Here also the genes associated with carbon metabolic pathways, energy metabolism, transport and amino acid metabolism had got down-regulated and this is now considered to be a key feature of the CSR [12, 22]. In contrast, out of the 423 DEGs in the DKO, only 133 DEGs were found to be down-regulated, of which 52 genes were common to both control and DKO (Fig. 3a). The major part of the total down-regulated genes belonged to the class of transporters (33 DEGs), energy metabolism (anaerobic) (15 DEGs) and cell motility (14 DEGs) (Fig. 2c). Unlike control, only a very limited number of down-regulated genes were found to be associated with key cellular processes, such as, translation (9 DEGs), central carbon metabolism (5 DEGs), carbohydrate metabolism (7 DEGs), transcription (4 DEGs), RNA & ribosome biogenesis (3 DEGs). Clearly the DKO was able to prevent the down-regulation of critical pathways which is the hallmark of a strong CSR. We next looked in more detail at the specific pathways which directly impact on recombinant protein yields.

### Respiratory metabolism

**Fig. 4:**
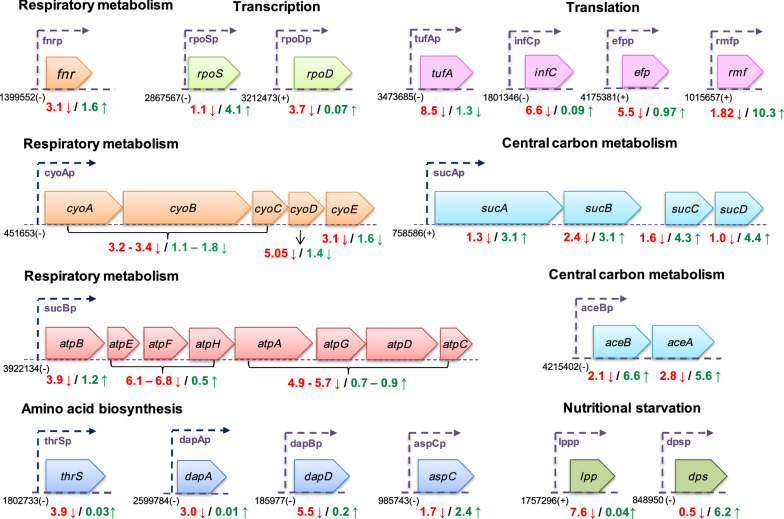
Critical genes which were found to be downregulated in control but not in the DKO strain. The numbers represent location of the transcriptional start site in the genome. ‘ + ’: upstream and ‘-’: downstream. The numbers below represent fold down-regulation (↓) or fold up-regulation (↑), Control: red; DKO: green

The respiratory metabolism of *E. coli* is efficient due to the fast kinetics of terminal oxidases [29]. It was observed that the global regulators *arcA* and *fnr* which regulate the expression of two major *E. coli* terminal oxidases: cytochrome bd-I (Cyd) and cytochrome bo9 (Cyo) [30] were 3.8-fold and 3.1-fold down-regulated in control compared to DKO strain where *arcA* was less down-regulated (1.42-fold) while *fnr* was up-regulated by 1.6-fold (Fig. 3b). Among terminal oxidases, Cyd is functional under micro-aerobic conditions due to its strong affinity to oxygen, whereas Cyo is dominant under fully aerobic conditions due to its low affinity to oxygen [31]. The *cyo* operon genes (*cyoABCDE*) were extensively down-regulated (3‒5-fold) in control, whereas the expression of *cydAB* genes was not significantly affected (Additional file 1: Table S1). However, in DKO, a much lower down-regulation of *cyoABCE* genes (1.1‒1.8-fold) and up-regulation of *cyoD* (1.45-fold) was observed (Fig. 4). Another important change in the DKO strain was seen in terms of unchanged transcript levels of the *atp* operon genes (encoding for *F0 F1- ATP synthase)* and *nuoA* gene (encoding the subunit A of NADH-quinone oxidoreductase) (Fig. 3b) in comparison to control, where these were severely down-regulated (3‒7-fold). Several researchers have shown that this down-regulation of energy metabolism genes post induction is a key feature of the CSR and a crucial factor behind the lowering of protein expression rates [14, 32]. These results demonstrate the ability of the DKO strain to exculpate the cell from experiencing this stress, thereby maintaining better homeostasis and retaining its capacity to generate the ATP required to meet the increased energy demands for recombinant protein synthesis.

### Transcription and translation

The gene expression levels of RNA polymerases *(rpoB, rpoC, rpoZ and rpoA)* which are essential for transcription initiation were found to be severely down-regulated (7‒10 fold) in control. However, in the DKO strain, only *rpoA* was down-regulated (2-fold) while the expression of the remaining polymerases stayed unaffected. Similarly, the transcript levels of *rpoD* encoding for the primary sigma factor 70 was unchanged in the DKO compared to a 3.7-fold down-regulation in control (Fig. 4). Since sigma factor 70 coordinates the transcription of house-keeping genes during exponential growth [33], its lack of impairment in the DKO ensured better cellular health and consequently improved expression capability of this strain.

The protein synthesis ability of *E. coli* is also determined by number of functional 70S ribosome units inside the cell. Transcriptomic studies showed a significantly higher down-regulation of 30S and 50S ribosomal genes in control, 8‒13-fold, compared to the DKO strain where it was only 1.1–3-fold (Fig. 3b) (Additional file 1: Table S1). Translation elongation factors that play an important role in bringing the aminoacyl-tRNA to the ribosome and facilitate the translocation of ribosome along the mRNA during protein synthesis [34] were also found to be less down-regulated in the DKO (1.2‒2.5-fold) compared to control (5.5‒10-fold) (Additional file 1: Table S1). These findings suggest that the effect of CSR on the transcriptional and translational machinery was much less pronounced in the DKO strain.

### Substrate uptake

Many transcriptomic studies have highlighted the negative effects of recombinant protein over-expression on nutrient uptake systems of *E. coli *[4, 35]. We also observed down-regulation of genes of glycerol catabolic regulon (up to 4-fold) in control. Interestingly this down-regulation of glycerol metabolism genes was intensified in the DKO strain (up to 8-fold) (Additional file 1: Table S1). Clearly this down-regulation of substrate metabolism genes in the DKO strain offset many of the gains obtained in terms of improved cellular health and expression capabilities and reflects the costs associated with tampering the finely tuned process of cellular dynamics that would have evolved to optimize cell survival.

### Cell motility

In *E. coli*, flagellar biosynthesis and motility is a tightly regulated process since it is energetically expensive [36]. Therefore, it is advantageous only when flagellar motility is required. It was observed that the flagellar genes belonging to *flgDEFGHIJK*, *fliAZ*, *fliDM*, *fliFGHIJK* operons were down-regulated up to 5.6-fold in the DKO strain, while they were up-regulated up to 6.2-fold in control (Fig. 3b) (Additional file 1: Table S1). This is possibly an associated evolutionary response to stress which is not only blocked but reversed in the DKO strain. The flagellar sigma factor *fliA* which was 2.8-fold up-regulated in control was also found to be 5.3-fold down-regulated in the DKO strain. This down-regulation of flagellar genes in DKO would have the added advantage of conserving and hence redirecting the energy expenditure of the cell towards recombinant protein synthesis.

### Analysis of up-regulated genes

Just as we had observed with the down-regulated genes, similarly a much smaller subset of genes was found to be up-regulated in the DKO; 290 DEGs compared to 896 DEGs in the control (Fig. 2b), clearly signifying a diminished CSR. The major component of the up-regulated genes in control belonged to the following categories; transporters (158 DEGs), carbohydrate metabolism (82 DEGs), amino acid metabolism (38 DEGs), cell motility (28 DEGs), energy metabolism (24 DEGs) and nucleotide metabolism (20 DEGs) (Fig. 2c). In the DKO strain, this list contained genes that mostly belonged to the following categories; central carbon metabolism (23 DEGs), transcription factors (10 DEGs), energy metabolism (14 DEGs), carbohydrate (28 DEGs) and transport (45 DEGs) (Fig. 2c). Apart from these, many other genes involved in protection against various kinds of stress were found to be up-regulated. These gene categories were analyzed in order to gain a better insight of cellular dynamics and their impact on recombinant protein synthesis.

### Central carbon metabolism

Transcriptomic analysis showed a differential up-regulation of several genes which are associated with central catabolic pathways. The data revealed a selective up-regulation of some TCA cycle genes (*sucABCD operon, sdhCDAB operon, icd, mdh*) only for the DKO strain (Fig. 3b) (Additional file 1: Table S1). The *sdhCDAB* operon of TCA cycle and *nuo* operon genes are known to be involved in aerobic electron transport chain to generate energy via oxidative phosphorylation [37, 38]. We also observed increased expression of *nuo* operon genes (2-fold) encoding the subunits of NADH dehydrogenase I in the DKO strain in contrast to their unchanged levels in control. There is a possible interconnectedness between the increased expression of such genes in the DKO with the higher rates of energy metabolism in terms of both ATP and reduction equivalents (NADH, NADPH & FADH) which together helped to meet the enhanced energy requirements imposed on these cells due to recombinant protein synthesis.

### Generalized stress response

The generalized stress response in *E. coli* is controlled by a global regulator ‘*rpoS’*, which is known to regulate the expression of 23% of *E. coli* genes under stress conditions [39, 40]. The DKO strain showed a 4-fold up-regulation of *rpoS* in contrast to a negligible change in control (Fig. 4). It was therefore no surprise to also observe the up-regulation of genes that are positively regulated by *rpoS *[41] (like *bfr, dps, osmB, osmC, osmY, psiF, uspB*) in the DKO strain compared to their down-regulated or unchanged expression in control (Additional file 1: Table S1). Some research groups have shown that *rpoS* also regulates the expression of *gadE* gene which is a transcriptional activator of glutamate-dependent acid resistance (GDAR) system [42]. In *E. coli*, GDAR plays an important role in maintaining cellular homeostasis under acidic conditions [43–45]. The transcriptomic data showed a much higher up-regulation of *gadE* regulated acid resistance genes i.e. *gadA, gadB, gadC* in the DKO strain (8‒10-fold) compared to control (3‒4-fold). This up-regulation could have boosted the general stress resistance of the DKO and allowed it to maintain homeostasis in spite of stress.

### Starvation stress

In *E. coli*, the stress response DNA binding protein ‘*dps*’ is an indicator of starvation stress inside cells [46, 47]. We found a 6-fold up-regulation of the *dps* gene in the DKO compared to its unchanged expression in control. Increased carbon starvation initiates a cascade of events inside the cell that results in release of carbon starvation proteins to prolong cell survival [48, 49]. It was observed that *cstA* gene encoding for a carbon starvation protein A that facilitates nutrient scavenging in terms of peptide transport and utilization [48] was 4-fold upregulated in DKO strain compared to its 1.4-fold up-regulation in control (Additional file 1: Table S1). The role of *cstA* gene in activating glycolysis and acetate metabolism in a CsrA dependent manner has also been studied [50]. An increased up-regulation for other carbon starvation inducible genes *csiD* (7.3-fold) [51, 52] and *slp* (starvation lipoprotein) (8.3-fold) was also observed in the DKO strain compared to control (*csiD* 1.76-fold, *slp* 3-fold). *slp* has been shown to promote cell survival during carbon starvation or stationary phase conditions [53]. It is quite remarkable that unlike the control the DKO strain was not only able to prevent but also anticipate the onset of stress and take remedial action by up-regulating global regulators like *rpoS* and *dps*.

### Amino acid biosynthesis

Amino acids play a crucial role in maintaining cellular metabolism and mediating the stress response. It is well established that their concentrations inside the cell affects gene expression, enzyme activities and redox homeostasis [54]. Transcriptomic analysis provided us an insight into the relative expression levels of genes associated with amino acid biosynthesis in both control and the DKO strain. However, these differences were not so evident since the use complex media for cultivation ensured an exogenous supply of amino acids, and this would have had a major impact on the results. A majority of the amino acid biosynthesis genes were found to be down-regulated by more than 2-fold in control, such as; *ilvNGAEDYC* (valine, leucine and isoleucine biosynthesis), *dapADF* (lysine biosynthesis), *aroFG* and *pheA* (aromatic amino acid biosynthesis), *aspC* (aspartate biosynthesis) and *thrCS* (threonine biosynthesis) (Figs. 3b and 4). However, the expression levels of most of these genes remained unchanged in the DKO except *ilvN* and *aspC* which were up-regulated by more than 2-fold (Additional file 1: Table S1). The genes for tryptophan (*trpE*) and cystiene biosynthesis (*cysH, cysI*) also remained 2‒4-fold up-regulated in both control and DKO strain. These findings suggest that the DKO strain is able to maintain a homeostatic environment by undergoing fewer changes in its amino acid biosynthetic pathways.

### Proteomic analysis

**Fig. 5:**
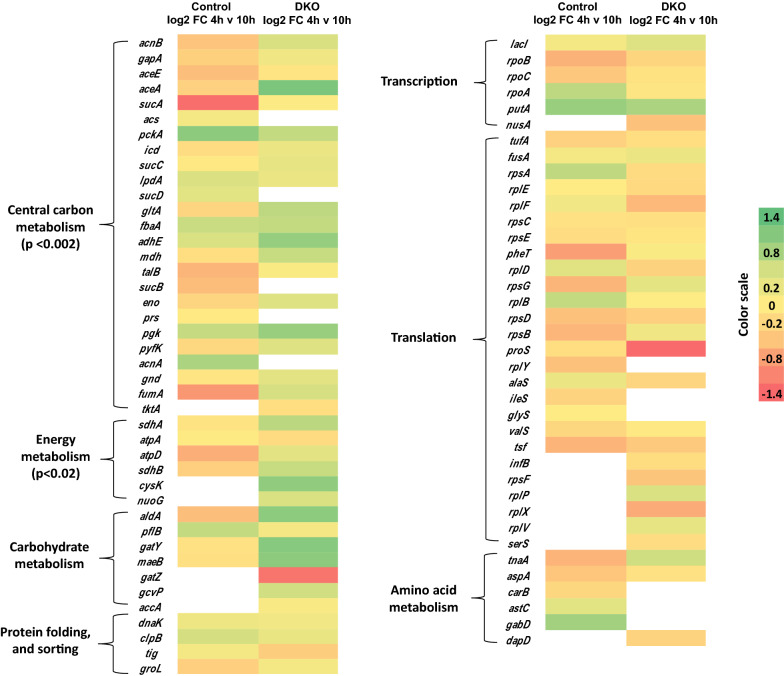
Heat maps of differentially expressed proteins obtained for control and DKO. The heat maps showing log2 fold change (4 h versus 10 h post induction) in expression levels of selected proteins in control and DKO strains belonging to following major categories: central carbon metabolism, translation, transcription, carbohydrate metabolism, energy metabolism, amino acid biosynthesis and protein folding & sorting. FC represents “fold change”. The white spaces represent those proteins which are absent either in control or DKO in top 100 list. The results for each functional category were analyzed using a Student’s *t-test* (Central carbon metabolism: **P < 0.002, Energy metabolism: *P < 0.02)

To observe the differential impact of the CSR on cellular health at the protein level, a preliminary study of the protein abundance profiles in the control and DKO strain was compared at the 4th and 10th h post induction. These time points were chosen since we had observed that both cell growth and protein expression capability remain unimpaired till the 4th h post induction after which it declines sharply in the control. We hypothesized that the CSR would significantly reduce the concentration of proteins which are critically required for protein synthesis in the control while its effect would be marginal in the DKO. We focused only on the top 100 most abundant proteins since their higher concentrations inside the cell allowed for a more precise quantitation by a label free LC MS/MS procedure. For normalization of protein content between samples, we used a multiplication factor so that the sum of the peak areas of the top 200 proteins obtained from LC–MS/MS analysis was equal between samples. These proteins were grouped into various categories like translation, central carbon metabolism, energy metabolism etc., similar to the categories used in our transcriptomic studies. We then calculated the log2 fold change for each protein across C4 and C10 and also between D4 and D10 (representing the 4th and 10th h post induction samples of the control and DKO respectively) (Additional file 1: Table S2). This was done only for those proteins which were present in the list of top 100 proteins at both time points. Figure 5 shows the heat map of this fold change in both the control and DKO for each group of proteins where it is clear that the central carbon metabolism and energy metabolism protein ratios for D10/D4 were far better than the C10/C4 ratios.

To estimate whether these differences were statistically significant we calculated the mean and variance of the log2(fold change) for each set of proteins belonging to the same category and performed a *t-test* (Additional file 1: Table S3). The results confirmed our previous observation that the proteins belonging to central carbon and energy metabolism were more abundant in the D10 sample which explained its superior ability to sustain recombinant protein expression. This was remarkable given that the D10 sample had accumulated a far higher amount of *L-asparaginase* (15% of the total cellular proteins) and MBP (13% of the total cellular proteins) leaving lesser space for host cell proteins inside the cell. This could possibly explain why the translational proteins were not significantly different in their ratios between the DKO and control even though the transcriptomic analysis showed a difference. The carbohydrate metabolism proteins did show a slightly higher level which was however not significant at the 95% confidence interval. This global analysis helped us identify the lumped changes in the protein abundance profiles which have a direct impact on cellular health and also validated the results of transcriptomic profiling. The results confirmed that the DKO was able to effectively block the cellular reprogramming which took place in the control which is why it was able to retain its expression abilities for a longer time period.

To conclude, the results of transcriptomic and proteomic analysis suggested that the DKO strain was able to substantially block the signaling pathways leading to the CSR and hence alleviate most of its negative impact on cellular metabolism. The absence of down-regulation of key pathway genes and their master regulators implied that the modified strain was able to maintain its energy pool, transcriptional and translational rates as well as carbon uptake and metabolism by preventing the reprogramming of its gene expression patterns which is otherwise triggered due to recombinant protein mediated cellular stress. The only downside of this knock out strategy was that it exacerbated the down-regulation of substrate uptake genes, which now remained the only bottleneck that could adversely impact on growth and protein expression capability.

### Growth and substrate utilization profiles of the DKO strain producing *L-asparaginase*

**Fig. 6:**
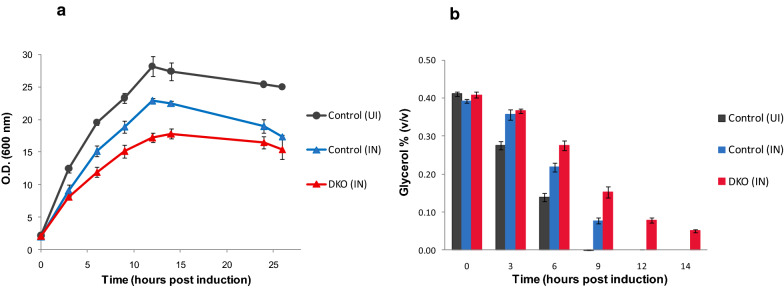
Growth and glycerol consumption profiles of control and DKO cells expressing *L-asparaginase.* Strains were cultivated in TB medium supplemented with 0.4% v/v glycerol and 10 mM MgSO_4_. Uninduced control (black), induced control (blue) and induced DKO strain (red). **a** Growth profile (O.D. at 600 nm) of control and DKO strain; and **b** Residual glycerol concentration in culture medium of control and DKO strain. Data represents mean ± SD of three independent biological replicates

To evaluate the phenotypic effect of this higher down-regulation of glycerol uptake genes, we compared the glycerol consumption profiles of the control and DKO strains expressing *L-asparaginase* in shake flask culture. An uninduced culture of the control strain was used as a benchmark to measure the normal growth and glycerol uptake capability of cells in the absence of cellular stress. Both induced cultures showed a decline in growth post induction, with the DKO strain displaying a sharper drop in growth rate (Fig. 6a) and a poorer glycerol uptake rate compared to control (Fig. 6b). Thus, control cells completely consumed the residual glycerol within 10 h post induction, while a significant amount of glycerol was leftover in the DKO culture even after 14 h post induction. Our preliminary studies on glycerol supplementation and it’s effect on growth rate revealed that 0.2% (v/v) glycerol is the optimum amount for shake flask studies, as it provides higher growth rate and reduces the problem of acetate accumulation in culture media (Additional file 1: Figure S4). Therefore, further studies were conducted by supplementing the TB media with 0.2% (v/v) glycerol.

Since poor substrate uptake would become a rate limiting factor for all cellular processes, we decided to supplement the DKO strain with additional copies of glycerol metabolism genes, and see its effect on the expression levels of recombinant protein. We therefore co-expressed glycerol kinase (*glpK)* and sn-glycerol-3-phosphate dehydrogenase (*glpD)* genes using the *pPROLAR.A122* vector backbone, since it was compatible with the plasmid used to express *L-asparaginase* in the DKO strain. Many previous studies have shown that these two genes play a critical role in enhancing glycerol uptake rates [20, 55–58].

### Co-expression of *glpDK* with *L-asparaginase*

The plasmid *pPROLAR.A122glpDK* carrying the genes (*glpK and glpD*) of the substrate utilization pathway was co-transformed along with *pMALS1Asp* into the DKO strain and labeled as the test strain. The DKO strain transformed only with the *pMALS1Asp* plasmid was used as a control for this study.

**Fig. 7:**
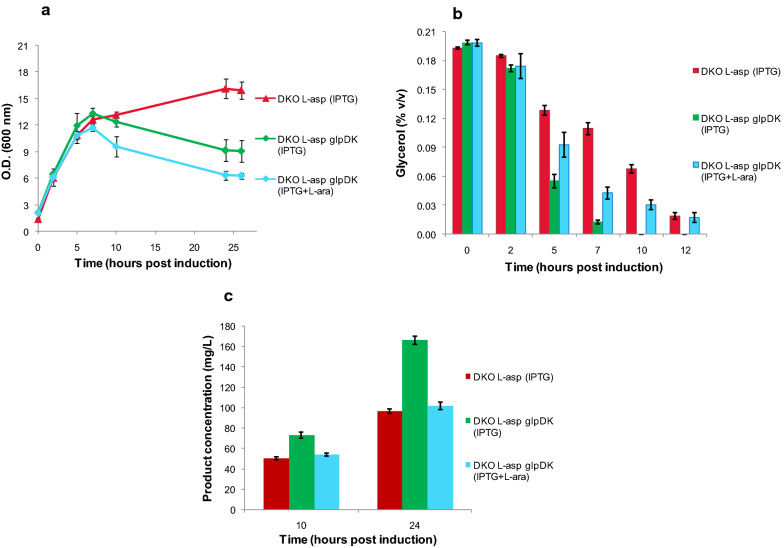
Growth, glycerol consumption and product concentration of DKO strain co-transformed with substrate utilization genes ‘*glpDK’*. The expression of recombinant *L-asparaginase* was tested in TB medium supplemented with 0.2% v/v glycerol and 10 mM MgSO_4_. The DKO strain transformed only with the *pMALS1Asp* plasmid (for *L-asparaginase* expression) was used as control (red). The test DKO cultures were co-transformed with both *pMALS1Asp* and *pPROLAR.A122glpDK* plasmids (for expression of substrate utilization genes *glpK* and *glpD*) but induced only for *L-asparaginase* expression (green), or induced for both plasmids (sky-blue). **a** Growth profiles of control and test (O.D. measurement at 600 nm); (**b**) Glycerol utilization profile; and (**c**) Product concentration (mg/L) at 10 h and 24 h, for control and test cultures. Data represents mean ± SD of three independent biological replicates

We had earlier observed that optimal supplementation of pathway related genes can be accomplished even without induction, since the leaky expression associated with plasmid based genes is enough to ensure an adequate supply of protein [59]. To confirm this, test cultures were either induced or left uninduced for *glpDK* expression while being induced for *L-asparaginase*. Interestingly we observed a higher decline in growth rates 7 h post induction for the test cultures compared to the control (Fig. 7a). This happened in spite of a superior glycerol uptake rate for both test cultures, indicating that the higher substrate consumption by cells was utilized primarily to enhance the flux towards product formation rather than growth (Fig. 7b). Also, the uninduced test culture performed significantly better in terms of both growth and product concentrations (Fig. 7c) demonstrating that the ideal supplementation levels of *glpDK* were achieved by simply allowing basal level expression of these genes.

**Fig. 8:**
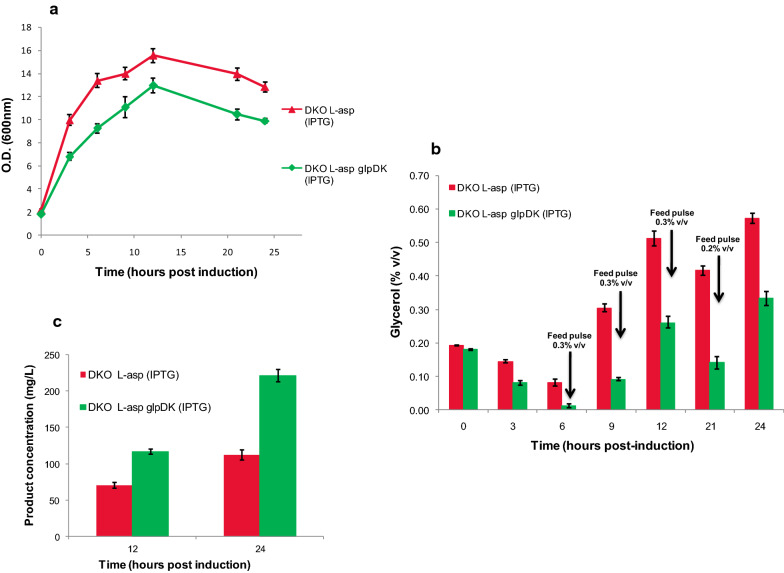
Effect of glycerol pulsing on glycerol uptake and product yield. Experiments were conducted in TB medium containing with 0.2% v/v glycerol and 10 mM MgSO_4._ The DKO strain transformed only with the *pMALS1Asp* plasmid (for *L-asparaginase* expression) was used as control (red), and DKO strain co-transformed with *pMALS1Asp* and *pPROLAR.A122glpDK* plasmids but induced only for *L-asparaginase* expression was used as test (green). **a** Growth profile of control and test cultures (O.D. measurements at 600 nm). **b** Glycerol utilization profile for control and test cultures upon continuous glycerol pulsing (0.3% v/v) at regular intervals of 3 h, starting from 6 h post induction till 12 h post induction. A final glycerol pulse (0.2% v/v) was added at 21 h post induction and (**c**) Product concentration (mg/L) at 12 h and 24 h for control and test cultures. The data represents mean ± SD of three independent biological replicates

Since the residual glycerol got completely exhausted within 7 h post induction, we decided to pulse the growing cultures with glycerol. Both control and test flasks were induced with IPTG at an O.D. of ~ 1.5–2. After 6 h post induction, a glycerol pulse was given to both control and test flasks and repeated every three hours till 12 h post induction. A final pulse was given 21 h post induction to check whether the cells retained their substrate uptake capability even after the onset of stationary phase. This higher availability of glycerol did not significantly alleviate the growth rate of the test culture but unlike the previous case no sharp fall in O.D._600_ was observed for the test flasks (Fig. 8a). Residual glycerol profiles showed that the substrate uptake capability of the test cultures remained high till 12 h post induction and then declined gradually (Fig. 8b) with the cells not being able to consume glycerol after the onset of stationary phase. The product concentration increased from being 1.72–fold higher to being 2.3–fold higher than control (Fig. 8c), underscoring the fact that the potential of this strain is truly realized when glycerol is available in the medium. Even though the production levels of *L-asparaginase* was significantly higher in this system, the functionality of the protein was preserved as was confirmed by estimating it’s specific activity which remained unchanged across both control and test samples (Additional file 1: Figure S5). It should be noted that the actual product formation ability per unit biomass was considerably greater for the test strain. Also the control used in this experiment was the DKO strain which had been previously shown to give >2-fold higher expression of *L-asparaginase* compared to the unmodified host [23], so the net improvement in yield over the unmodified host was much higher.

## Conclusions

The results validate our hypothesis that *elaA* and *cysW* are part of the signaling network that triggers the onset of the CSR in *E. coli*. This CSR acts on multiple pathways and effectively reprograms the complete cellular machinery leading to the feedback inhibition of recombinant protein synthesis. Upstream interventions that block the initiation of this CSR are far more powerful and elegant tools to counter this effect and can open up exciting avenues for the design of next generation platforms for recombinant protein expression. The work also demonstrates the need for a more nuanced and context dependent view of regulatory hierarchies within the cell. Thus, genes which under normal conditions occupy the bottom rung of the regulatory map of *E. coli* (EcoCyc) and have no known downstream regulates, can in the presence of stress suddenly become critically important and impact on the expression of hundreds of genes. This work would also spur the exploration of signaling pathways that regulate heterologous protein synthesis in *E. coli* leading to a more comprehensive understanding of cellular dynamics.

## Materials and methods

### Strains and plasmids

*E. coli* strain BW25113 was obtained from Yale CGSC (The Coli Genetic Stock Center), USA. The double knockout strain ‘BW25113*ΔelaAΔcysW’* and the recombinant plasmid *pMALS1Asp* were previously developed in our lab [18, 23]. The *sfGFP*-*pBAD* plasmid was taken from Addgene (Addgene plasmid # 54,519). *pBAD-Rubella-sfGFP* plasmid containing Rubella E1-*sfGFP* expression cassette was developed in our lab as a part of this study.

### Clone construction

#### Construction of Rubella E1-*sfGFP* expression cassette

Both Rubella E1 glycoprotein (789 bp) and *sfGFP* genes (720 bp) were PCR amplified using primers (Table 1), which also introduced a sequence for a flexible linker (Gly-Gly-Gly-Gly-Ser) peptide between them. The sequence encoding the flexible linker peptide was present in the overlapping ends of the reverse primer for E1 glycoprotein gene & forward primer of the *sfGFP* gene. The *pBAD24* expression vector backbone was also PCR amplified. The resultant PCR amplified gene products were inserted downstream of *araBAD* promoter in a *pBAD24* expression vector using the principle of homologous recombination described by Jacobus and Gross [60] (Additional file 1: Figure S6(A-C)). Transformants grown on respective antibiotic plates were screened by colony PCR and confirmed by restriction digestion (Additional file 1: Figure S6(D-E)).

**Table 1:** List of primers used for cloning

S.No	Name	Sequence (5′ → 3′)
Primers used for cloning of *Rubella-sfGFP*		
1	*Rubella Fw* + *overhang*	GAGATATACATATGCATATGCTGTCAGTCGCAGGCGT
2	*Rubella Rv* + *overhang*	CACAGCCGTGGTATGCGTACCCCGCATAGATCTCGATCGAGAACCACCACCACC
3	*sfGFPFw* + *overhang*	ACGGCTGTGGGTGGTGGTGGTTCTCGATCGAGATCTATGCGGGGTTCTCATCATCA
4	*sfGFPRv* + *overhang*	TTACTTGTACAGCTCGTCCATGTGCCTGCAGGTCGACTCTAG
5	*Vector Fw* + *overhang*	TGGACGAGCTGTACAAGTAACTAGAGTCGACCTGCAGGCATGCA
6	*Vector Rv* + *overhang*	ACGCCTGCGACTGACAGCATATGCATATGTATATCTC
Primers used for cloning of glycerol metabolism genes		
1	*FW glpDvec overhang*	TGGAGATGACGATGACAAGGTGGTCGACAAGCTTATGGAAACCAAAGATCTGATTGT GATAGG
2	*RV glpDara overhang*	CGCTAATCTTATGGATAAAAATGCTATGCTCGATTACGACGCCAGCGATAACCTCT
3	*FW AraglpD overhang*	TATACGCAGCAGAGGTTATCGCTGGCGTCGTAATCGAGCATAGCATTTTTATCCATA AGATTAG
4	*RV AraglpK overhang*	TGGTCGAGCGCAACGATATATTTTTTTTCAGTCATGGGTACCTTTCTCCTCTTTAATG AATTCT
5	*FW glpKara overhang*	TCACACAGAATTCATTAAAGAGGAGAAAGGTACCCATGACTGAAAAAAAATATATCG TTGCGCTC
6	*RV glpKvec overhang*	CCGCATCGATCGGGCCCTGAGGCCTGCAGGGATCCTTATTCGTCGTGTTCTTCCCA CGC
7	*FW vecglpK overhang*	AACGCGCGATGGCGTGGGAAGAACACGACGAATAAGGATCCCTGCAGGCCTCAGG GCC
8	*RV vecglpD overhang*	CGCCCCCTATCACAATCAGATCTTTGGTTTCCATAAGCTTGTCGACCACCTTGTCAT CGTCATCTC
9	*glpKFw*	CCAAGCTTATGACTGAAAAAAAATATATCG
10	*glpKRv*	GCTCTAGATTATTCGTCGTGTTCTTC
11	*glpDFw*	GCAAGCTTATGGAAACCAAAGATCTGATTG
12	*glpDRv*	AATCTAGATTACGACGCCAGCGATAACC

#### Construction of expression vector *glpDK*

Both *glpD* and *glpK* genes were cloned in the same plasmid system *pPROLAR.A122* by introducing a tandem *P*_*lac/ara1*_ promoter region into the MCS of this vector. For this, the vector and inserts were linearized by PCR using primers that contained 30–35 bp overhangs (listed in Table 1) matching the ends of the gene fragments to be cloned in respective directions (Additional file 1: Figure S7(A)). The amplified gene products were digested with *DpnI*, eluted from 1% agarose gel and co-transformed into *E. coli DH5α* cells in a vector insert ratio of 2:1 using the method of homologous recombination (Additional file 1: Figure S7(B-C)). Positive clones were screened by colony PCR using forward *glpD* and reverse *glpK* primers (Additional file 1: Figure S7(D)) and the plasmid was labeled as *pPROLAR.A122glpDK.*

### Cell culture

Cells were grown in a microbioreactor (BioLector, m2P labs GmbH, Germany) which provides a broad range of defined oxygen transfer rates (OTR) [61]. The fermentation parameters i.e. biomass, pH, dissolved oxygen (DO) and fluorescence were monitored online. We used Terrific broth (TB) (yeast extract 24 g/L, tryptone 12 g/L, potassium dihydrogen phosphate 2.2 g/L, dibasic potassium phosphate 9.4 g/L, pH 7.2) (HiMedia Laboratories) containing 0.4% (v/v) glycerol for our studies. Both control (BW25113) and DKO (BW25113*ΔelaAΔcysW*) strains freshly transformed with *pMALS1Asp* plasmid were inoculated in 10 ml TB media containing 100 μg/ml ampicillin and grown overnight. These primary cultures were used to inoculate secondary cultures containing 50 ml TB media having the same antibiotic concentration. This was grown further for 8 h (O.D. ~ 9‒10) and then used as inoculum (2% of the final media volume) for the microbioreactor studies. The experiment was performed in 48 well FlowerPlate and each well contained 1 ml of TB media supplemented with 10 mM MgSO_4_ & 0.4% (v/v) glycerol. Temperature was set at 37 °C and the DO monitoring showed that it was ≥30% throughout the cultivation period due to rigorous shaking at 1400 rpm. After ~ 2–2.5 h when cells reached mid-exponential phase, cultures were induced by 1 mM IPTG (final concentration) for *L-asparaginase* expression. The experiments were done in a batch mode in biological triplicates.

### Transcriptomic analysis

Microbioreactor experiments were done in batch mode for both control and DKO strains producing *L-asparaginase* as described in the previous section. 900µl samples were harvested at the 0th and 6th h post induction. 0th h (uninduced) samples were taken as a control for every run. To stop mRNA degradation, 100μL of ice cold EtOH/Phenol stop solution (5% water saturated phenol (pH < 7.0) in ethanol) was immediately added to the 900μL culture. The culture was centrifuged at 8000 rpm for 2 min at 4 ℃ and RNA isolation was done from sample pellets. Samples were processed for removal of genomic DNA using *DNase I* (Thermo Fisher Scientific, USA) treatment. The total RNA concentration and its quality were determined by an Agilent 2100 Bioanalyzer by looking at its RNA Integrity Number (RIN). RNA samples which had a RIN number > 7 (on a scale of 1–10) were used for further processing. The RNA samples were sent to AgriGenome (Cochin, India) for further library preparation & RNA sequencing. The detailed protocol for RNA-seq data analysis is given in Additional file 1: Method S8. The raw reads and the processed data files have been deposited in the NCBI’s GEO Database and are accessible through GEO series accession number GSE108442.

### Quality check for RNA seq data

The quality check for the data generated by Illumina HiSeq 2500 platform (RNA Seq technology) was performed by plotting logCPM (reads count per million) values for two biological replicates belonging to the same time-point. RNA sequencing for 6th h post induction sample of control was done in biological duplicates. We found a fair degree of correlation between these replicates with the correlation coefficient being > 0.8 (Additional file 1: Figure S9), indicating a good quality of the generated RNA Seq data.

### Quantitative real time PCR (qRT-PCR)

Real time PCR analysis was performed to validate the results of RNA sequencing. The detailed protocol for qRT-PCR is given in Additional file 1: Method S10. The relative quantification was done using 2^−ΔΔCT^ method [62] and fold change values were used to calculate log2 fold change. The primer sequences used for the amplification of specific genes are listed in Additional file 1: Table S11.

### RT-PCR validation of RNA seq data

To experimentally validate the genes expression levels obtained from RNA seq data analysis, we plotted the qPCR expression levels (log2 scale) of randomly selected eight genes against their expression levels (log2 scale) obtained from RNA seq analysis. The results showed that the genes expression profiles obtained by qRT-PCR were mostly consistent with those measured by RNA seq analysis (Additional file 1: Figure S12). The fold change values of qRT-PCR and RNA-Seq showed a strong positive correlation with R^2^ = 0.884 for control and R^2^ = 0.818 for DKO. These results demonstrate the credibility of RNA-Seq data generated in this study.

### Proteomic analysis

For proteome analysis, 20 μg of the total cell protein was isolated from 4th and 10th h post induction cultures of control and DKO strains and subsequently subjected to reduction and alkylation of disulfide bonds with 10 mM dithiothreitol (DTT) at 65°C for 5 min followed by 40 mM iodoacetamide for 1 h in dark at room temperature. Digestion was performed using sequencing grade trypsin (1:50, enzyme: total protein) (Promega Corporation, USA) overnight at 37°C and the reaction was stopped using 0.1% formic acid. The tryptic digests were desalted and concentrated using ZipTip (Pierce C18 Tips, Thermo). The resultant peptides were acidified with 0.1% formic acid and analyzed with an Orbitrap Velos Pro mass spectrometer coupled with a Nano-LC 1000 system (Thermo Fisher Scientific). The detailed protocol for label free LC MS/MS protein quantification is given in Additional file 1: Method S13.

### Statistical analysis

The statistically significant differences between the transcriptome of the control and DKO was calculated using paired *t-test* performed on log2 fold change in gene expression levels (0 h versus 6 h) of genes categorized under different functional categories. Statistical analysis was performed using GraphPad Prism Software (version 5, GraphPad Software Inc, San Diego, CA, USA) (Additional file 1: Table S14). For the proteomic analysis we first calculated the log2 fold change in protein levels between the two time points (4th and 10th hour post induction) in both control and DKO strain. A lumped estimate of the mean values of this change for each protein category was done using this log transformed data. The difference between the means of the control and DKO was then statistically analyzed using a *t-test* for unequal variances (Additional file 1: Table S3). *p* ≤ 0.05 was considered as statistically significant.

### Glycerol and *L-asparaginase* measurement

Residual glycerol concentration in the culture supernatant was measured using Agilent HPLC 1260 Infinity system (Agilent Technologies Inc., USA) equipped with a refractive index detector. AMINEX HPX-87H column (7.8 × 300 mm) (BioRad Laboratories) was used for this purpose. Mobile phase consisted of 5 mM H_2_SO_4_ with a flow rate of 0.5 mL/min. Column temperature was kept at 50°C. The culture supernatant was filtered with a 0.22 µm syringe filter before injection. *L-asparaginase* activity was measured using a protocol described earlier [63].

## Supplementary Information

**Additional file 1: Table S1.** Log2 fold change in expression levels of genes belonging to different functional categories in control and double knockout (DKO) strains. **Table S2.** Log2 fold change in expression levels of proteins (4 h versus 10 h post induction) belonging to different functional categories in control and double knockout (DKO) strains. **Table S3:.** Statistical analysis of protein expression data for control and double knockout (DKO) strains. **Figure S4.** (A) Growth profile of *E. coli* BW25113 strain growing in TB media containing no glycerol, 0.2% and 0.4% (v/v) glycerol. (B) Glycerol consumption profiles of control and DKO strain growing in TB media containing 0.2% (v/v) glycerol. **Figure S5.** Confirmation of preserved functionality of *L-asparaginase* by estimating its specific activity. **Figure S6.** Cloning of Rubella E1 glycoprotein (target gene) and *sfGFP* into the *pBAD24* expression vector in *E. coli* DH5α strain using the principle of homologous recombination. **Figure S7.** (A & B) Pictorial representation of construction of expression vector *pPROLAR.A122glpDK* (5.9 kb). (C) Gel picture showing amplification of *glpD* gene, *Ara* promoter, *glpK* gene and vector backbone (D) PCR confirmation of *glpD-araProm-glpK* insert. **Method S8.** RNA-seq analysis procedure. **Figure S9.** Correlation plot between log CPM values of two biological replicates of control strain (6 h post induction) expressing *L-asparaginase*. **Method S10.** qRT-PCR protocol. **Table S11.** List of primers used for qRT-PCR. **Figure S12.** Correlation analysis of log2 fold change values obtained from RNA-Seq and qRT-PCR. **Method S13.** Label free LC MS/MS protein quantification. **Table S14.** Statistical analysis of gene expression data obtained from RNA seq analysis for control and double knockout (DKO) strains.

## Data Availability

The RNA-seq data generated during this study is available the Gene Expression Omnibus database at national center for biotechnology information (NCBI) under the with accession number GSE108442 (GEO: https://www.ncbi.nlm.nih.gov/geo/query/acc.cgi?acc=GSE108442). All other data generated or analyzed during this study are included in this article and its Additional file 1.

## Abbreviations

CSR: Cellular stress response
DEGs: Differentially expressed genes
DKO: Double knockout
sfGFP: Super-folding green fluorescent protein
TB: Teriffic broth
HPLC: High pressure liquid chromatography
