# Supplementary material for: Designing next generation recombinant protein expression platforms by modulating the cellular stress response in Escherichia coli
Source: Microb Cell Fact. 2020 Dec 11;19:227. doi: 10.1186/s12934-020-01488-w (PMC7730785; doi:10.1186/s12934-020-01488-w)
Supplement: Supplementary file 1 — Additional file 1: Table S1. Log2 fold change in expression levels of genes belonging to different functional categories in control and double knockout (DKO) strains. Table S2. Log2 fold change in expression levels of proteins (4 h versus 10 h post induction) belonging to different functional categories in control and double knockout (DKO) strains. Table S3:. Statistical analysis of protein expression data for control and double knockout (DKO) strains. Figure S4. (A) Growth profile of E. coli BW25113 strain growing in TB media containing no glycerol, 0.2% and 0.4% (v/v) glycerol. (B) Glycerol consumption profiles of control and DKO strain growing in TB media containing 0.2% (v/v) glycerol. Figure S5. Confirmation of preserved functionality of L-asparaginase by estimating its specific activity. Figure S6. Cloning of Rubella E1 glycoprotein (target gene) and sfGFP into the pBAD24 expression vector in E. coli DH5α strain using the principle of homologous recombination. Figure S7. (A & B) Pictorial representation of construction of expression vector pPROLAR.A122glpDK (5.9 kb). (C) Gel picture showing amplification of glpD gene, Ara promoter, glpK gene and vector backbone (D) PCR confirmation of glpD-araProm-glpK insert. Method S8. RNA-seq analysis procedure. Figure S9. Correlation plot between log CPM values of two biological replicates of control strain (6 h post induction) expressing L-asparaginase. Method S10. qRT-PCR protocol. Table S11. List of primers used for qRT-PCR. Figure S12. Correlation analysis of log2 fold change values obtained from RNA-Seq and qRT-PCR. Method S13. Label free LC MS/MS protein quantification. Table S14. Statistical analysis of gene expression data obtained from RNA seq analysis for control and double knockout (DKO) strains. [file 12934_2020_1488_MOESM1_ESM.pdf]

Supplementary information

Designing next generation recombinant protein expression platforms by modulating the cellular stress response in *Escherichia coli*

Richa Guleria[1,#], Priyanka Jain[1,#], Madhulika Verma[2] and Krishna J. Mukherjee[1,3,*]

[1]School of Biotechnology, Jawaharlal Nehru University, New Delhi, 110067, India

[2]School of Computational and Integrative Sciences, Jawaharlal Nehru University, New Delhi, 110067, India

[3]Department of Biochemical Engineering and Biotechnology, Indian Institute of Technology Delhi, New Delhi, 110016, India

[#]Authors equally contributed to this work

***Correspondence:** Krishna J. Mukherjee, School of Biotechnology, Jawaharlal Nehru University, New Delhi, 110067, India.

Phone: (+91) 011 26704089

Email: kjmukherjee@jnu.ac.in / kjmukherjee@dbeb.iitd.ac.in

This additional file 1 contains:

Tables S1, S2, S3, S11, S14

Figures S4, S5, S6, S7, S9, S12

Methods S8, S10, S13

Table S1: Log2 fold change in expression levels of genes belonging to different functional categories in control and double knockout (DKO) strains.

Gene	Gene product	Gene expression (log 2 Fold Change)	
		Control	DKO
**Energy metabolism**			
sdhA	Succinate dehydrogenase flavoprotein subunit	1.41	3.35
sdhB	Succinate dehydrogenase iron-sulfur subunit	0.51	2.86
sdhC	Succinate dehydrogenase cytochrome b556 subunit	0.19	3.13
sdhD	Succinate dehydrogenase hydrophobic membrane anchor subunit	0.75	3.48
nuoJ	NADH-quinone oxidoreductase subunit J	1.03	2.24
nuoL	NADH-quinone oxidoreductase subunit L	0.61	2.01
nuoI	NADH-quinone oxidoreductase subunit I	0.45	2.18
nuoH	NADH-quinone oxidoreductase subunit H	0.09	2.29
nuoK	NADH-quinone oxidoreductase subunit K	0.50	2.10
nuoG	NADH-quinone oxidoreductase subunit G	0.62	1.91
nuoA	NADH-quinone oxidoreductase subunit A	-3.03	0.49
ndh	NADH:quinone oxidoreductase II	-1.74	-2.76
cyoE	heme o synthase	-3.10	-1.63
cyoB	cytochrome bo3 ubiquinol oxidase subunit 1	-3.23	-1.63
cyoC	cytochrome bo3 ubiquinol oxidase subunit 3	-3.26	-1.82
cyoA	cytochrome bo3 ubiquinol oxidase subunit 2	-3.47	-1.13
atpB	ATP synthase Fo complex subunit a	-3.96	-1.24
atpD	ATP synthase F1 complex subunit beta	-4.90	0.98
cyoD	cytochrome bo3 ubiquinol oxidase subunit 4	-5.05	1.46
atpG	ATP synthase F1 complex subunit gamma	-5.09	0.96
atpC	ATP synthase F1 complex subunit epsilon	-5.71	0.96
atpA	ATP synthase F1 complex subunit alpha	-5.79	0.75
atpE	ATP synthase Fo complex subunit c	-6.18	0.56
atpH	ATP synthase F1 complex subunit delta	-6.66	0.89

atpF	ATP synthase Fo complex subunit b	-6.82	0.59
**Transcription**			
rpoA	RNA polymerase subunit alpha	-10.95	-2.01
rpoB	RNA polymerase subunit beta	-8.60	-0.95
rpoC	RNA polymerase subunit beta'	-8.73	-1.33
rpoZ	RNA polymerase subunit omega	-6.65	-0.98
rpoS	RNA polymerase, sigma S (sigma 38) factor	-1.15	4.11
rpoD	RNA polymerase, sigma 70 (sigma D) factor	-3.71	0.07
rpoE	RNA polymerase sigma E factor b4725	-4.67	-0.92
rpoH	RNA polymerase, sigma H (sigma 32) factor	2.91	0.83
rpoN	RNA polymerase, sigma N (sigma 54) factor	-2.29	0.17
**Ribosomal genes**			
rpsA	30S ribosomal subunit protein S1	-8.67	-2.12
rpsB	30S ribosomal subunit protein S2	-9.03	-2.64
rpsC	30S ribosomal subunit protein S3	-10.39	-3.07
rpsD	30S ribosomal subunit protein S4	-11.62	-1.78
rpsE	30S ribosomal subunit protein S5	-10.72	-1.69
rpsF	30S ribosomal subunit protein S6	-9.55	-2.06
rpsG	30S ribosomal subunit protein S7	-9.53	-1.69
rpsH	30S ribosomal subunit protein S8	-10.43	-1.24
rpsI	30S ribosomal subunit protein S9	-8.56	-2.60
rpsJ	30S ribosomal subunit protein S10	-11.09	-2.16
rpsK	30S ribosomal subunit protein S11	-9.79	-1.60
rpsL	30S ribosomal subunit protein S12	-9.45	-1.54
rpsM	30S ribosomal subunit protein S13	-10.68	-1.44
rpsN	30S ribosomal subunit protein S14	-11.62	-1.14
rpsO	30S ribosomal subunit protein S15	-10.29	-2.37
rpsP	30S ribosomal subunit protein S16	-10.35	-1.61
rpsQ	30S ribosomal subunit protein S17	-13.15	-3.03

rpsR	30S ribosomal subunit protein S18	-7.81	-1.93
rpsS	30S ribosomal subunit protein S19	-9.80	-2.96
rpsT	30S ribosomal subunit protein S20	-10.96	-2.95
rplA	50S ribosomal subunit protein L1	-11.58	-1.54
rplB	50S ribosomal subunit protein L2	-10.52	-2.77
rplC	50S ribosomal subunit protein L3	-10.18	-2.37
rplD	50S ribosomal subunit protein L4	-10.26	-2.52
rplE	50S ribosomal subunit protein L5	-10.56	-1.02
rplF	50S ribosomal subunit protein L6	-11.01	-1.37
rplI	50S ribosomal subunit protein L9	-9.21	-1.95
rplJ	50S ribosomal subunit protein L10	-12.22	-2.43
rplK	50S ribosomal subunit protein L11	-11.55	-1.49
rplL	50S ribosomal subunit protein L12	-12.89	-2.75
rplM	50S ribosomal subunit protein L13	-9.04	-2.46
rplN	50S ribosomal subunit protein L14	-10.75	-1.27
rplO	50S ribosomal subunit protein L15	-10.22	-1.98
rplP	50S ribosomal subunit protein L16	-10.12	-3.09
rplQ	50S ribosomal subunit protein L17	-11.35	-2.23
rplR	50S ribosomal subunit protein L18	-10.14	-1.66
rplS	50S ribosomal subunit protein L19	-11.38	-1.56
rplT	50S ribosomal subunit protein L20	-10.16	-1.84
rplU	50S ribosomal subunit protein L21	-8.96	-2.24
rmf	ribosome modulation factor	-1.82	10.35
**Translation elongation factors**			
tufA	translation elongation factor Tu 1	-8.56	-1.38
tufB	translation elongation factor Tu 2	-10.14	-1.4
tsf	protein chain elongation factor EF-Ts	-9.55	-2.51
fusA	elongation factor G	-8.7	-1.98
efp	protein chain elongation factor EF-P	-5.51	0.97

infA	translation initiation factor IF-1	-5.57	-2.51
infB	translation initiation factor IF-2	-4.35	-1.11
infC	translation initiation factor IF-3	-6.65	0.99
**Global Regulators**			
arcA	aerobic respiration control protein ArcA	-3.83	-1.42
fnr	Fumarate and nitrate reduction regulatory protein	-3.10	1.67
gadX	DNA-binding transcriptional dual regulator or glutamate-dependent acid resistance regulator	0.43	3.69
**Substrate uptake**			
glpA	anaerobic glycerol-3-phosphate dehydrogenase subunit A	-3.55	-9.12
glpB	anaerobic glycerol-3-phosphate dehydrogenase subunit B	-1.28	-8.09
glpF	glycerol facilitator	-3.17	-2.75
glpC	anaerobic glycerol-3-phosphate dehydrogenase subunit C	3.37	-5.96
glpK	glycerol kinase	-3.30	-2.74
glpD	aerobic glycerol 3-phosphate dehydrogenase	-4.05	-4.84
glpT	sn-glycerol 3-phosphate:phosphate antiporter	-2.66	-5.93
**Nutrient starvation**			
slp	starvation lipoprotein	3.07	8.34
csiD	glutarate dioxygenase	1.76	7.32
cstA	carbon starvation protein A	1.38	4.08
lpp	murein lipoprotein	-7.64	-0.04
dps	starvation-inducible DNA-binding protein	-0.58	6.21
**Central carbon metabolism**			
aceA	isocitrate lyase	-2.13	6.66
aceB	malate synthase A	-2.88	5.69
poxB	pyruvate oxidase	2.79	4.80
pta	phosphate acetyltransferase	-4.06	-1.28
acs	acetyl-CoA synthetase	4.30	5.41
sucA	2-oxoglutarate dehydrogenase E1 component	-1.31	3.11
sucB	2-oxoglutarate dehydrogenase E2 component	-2.46	3.12

sucC	succinyl-CoA synthetase beta subunit	-1.63	4.38
sucD	succinyl-CoA synthetase alpha subunit	-1.01	4.40
icd	isocitrate dehydrogenase	-3.20	2.82
mdh	malate dehydrogenase	-3.52	3.54
**Amino acid biosynthesis**			
ilvN	acetolactate synthase I/III small subunit	-3.09	2.59
ilvG	Pseudogene	-5.39	-1.18
ilvA	threonine deaminase	-2.98	0.98
ilvE	branched-chain amino acid aminotransferase	-2.98	0.76
ilvD	dihydroxy-acid dehydratase	-2.37	0.76
ilvY	DNA-binding transcriptional dual regulator IlvY	-2.07	0.70
ilvC	ketol-acid reductoisomerase	-1.98	0.58
dapA	4-hydroxy-tetrahydrodipicolinate synthase	-3.01	0.01
dapB	4-hydroxy-tetrahydrodipicolinate reductase	-1.17	0.37
dapD	tetrahydrodipicolinate succinylase	-5.52	0.27
dapF	diaminopimelate epimerase	-4.02	0.42
aroF	3-deoxy-7-phosphoheptulonate synthase	-2.03	0.89
aroG	3-deoxy-7-phosphoheptulonate synthase	-1.56	1.34
pheA	chorismate mutase / prephenate dehydratase	-2.47	1.48
tyrB	tyrosine aminotransferase	-1.47	-1.27
trpE	anthranilate synthase subunit TrpE	4.73	3.73
cysH	phosphoadenosine phosphosulfate reductase	4.24	1.98
cysI	sulfite reductase, hemoprotein subunit	4.00	2.61
glyA	serine hydroxymethyltransferase	-3.15	-0.48
aspC	aspartate aminotransferase	-1.75	2.47
thrB	homoserine kinase	-1.75	1.20
thrC	threonine synthase	-2.07	0.32
thrS	threonine--tRNA ligase	-3.94	0.03
thrA	fused aspartate kinase/homoserine dehydrogenase 1	-1.38	1.53

metL	fused aspartate kinase/homoserine dehydrogenase 2	-2.00	0.74
hisG	ATP phosphoribosyltransferase	1.27	-0.55
**Cell motility**			
flgA	flagellar basal body P-ring formation protein	2.97	-2.82
flgD	flagellar biosynthesis, initiation of hook assembly	3.62	-5.61
flgE	flagellar hook protein	3.44	-4.80
flgF	flagellar basal-body rod protein	4.20	-4.68
flgG	flagellar basal-body rod protein	4.74	-3.91
flgI	flagellar P-ring protein	5.12	-3.43
flgJ	putative peptidoglycan hydrolase	5.67	-2.56
flgK	flagellar hook-filament junction protein 1	4.83	-1.31
fliA	RNA polymerase, sigma 28 (sigma F) factor	2.81	-5.31
fliD	flagellar filament capping protein	6.22	-2.58
fliF	flagellar basal-body MS-ring and collar protein	4.51	-5.17
fliG	flagellar motor switch protein	2.58	-4.12
fliH	flagellar biosynthesis protein	4.92	-3.91
fliK	flagellar hook-length control protein	5.05	-2.51
fliM	flagellar motor switch protein	3.72	-4.20
fliZ	DNA-binding transcriptional regulator	3.27	-2.36
**Stress resistance**			
gadA	glutamate decarboxylase A	3.27	8.24
gadB	glutamate decarboxylase B	4.63	10.67
gadC	L-glutamate:4-aminobutyrate antiporter	3.47	10.41
bfr	Bacterioferritin	-2.25	5.81
osmB	osmotically-inducible lipoprotein	2.13	6.27
osmC	osmotically inducible peroxiredoxin	1.85	6.21
osmY	periplasmic chaperone	1.30	5.68
psiF	Phosphate starvation-inducible protein	-0.06	3.08
uspB	universal stress protein B	0.82	3.59

Table S2: Log2 fold change in expression levels of proteins (4 h versus 10 h post induction) belonging to different functional categories in control and double knockout (DKO) strains. The log2FC (4 h v 10 h) values for proteins that are absent either in control or DKO in top 100 list are left blank.

**Functional category**	**Protein**	**Control**	**DKO**
		**log2FC 4h vs 10h**	**log2FC 4h vs 10h**
**Central carbon metabolism**	***acnB***	-0.4512	0.3080
	gapA	-0.3358	0.0828
	aceE	-0.5168	-0.1238
	aceA	-0.3224	1.1814
	sucA	-1.3383	-0.0298
	acs	0.0449	
	pckA	1.0114	0.5104
	icd	-0.2069	0.1350
	sucC	-0.0856	0.1670
	lpdA	0.2873	0.1308
	sucD	0.2216	
	gltA	-0.2925	0.5581
	fbaA	0.4467	0.5047
	adhE	0.2959	0.9308
	mdh	-0.1837	0.4653
	talB	-0.6075	-0.0007
	sucB	-0.5299	
	eno	-0.2869	0.2490
	prs	-0.0762	
	pgk	0.4799	0.8874
	pyfK	-0.2483	0.2465
	acnA	0.7226	
	gnd	-0.1290	0.2085
	fumA	-0.9183	0.3324
	tktA		-0.1907
**Energy metabolism**	***sdhA***	-0.1362	0.5800
	atpA	-0.0613	-0.2315
	atpD	-0.6804	0.2035
	sdhB	-0.3414	0.4716
	cysK		0.9755
	nuoG		0.2959
**Carbohydrate metabolism**	***aldA***	-0.5036	1.0089
	pflB	0.4966	0.0414
	gatY	-0.1577	1.0641

	maeB	-0.1767	1.0121
	gatZ		-1.2707
	gcvP		0.3695
	accA		0.0162
**Protein folding and sorting**	***dnaK***	0.1076	0.0773
	clpB	0.3423	0.1504
	tig	0.0522	-0.3671
	groL	-0.3394	0.0478
**Transcription**	***lacI***	0.0555	0.2481
	rpoB	-0.6442	-0.2833
	rpoC	-0.4236	-0.1447
	rpoA	0.5589	-0.1118
	putA	0.8967	0.7195
	nusA		-0.4809
**Translation**	***tufA***	-0.3173	-0.1892
	fusA	0.0540	0.1368
	rpsA	0.5320	-0.2319
	rplE	-0.0558	-0.2421
	rplF	0.0788	-0.5694
	rpsC	-0.1548	-0.1652
	rpsE	-0.2069	-0.1159
	pheT	-0.8487	0.0061
	rplD	0.2240	-0.3108
	rpsG	-0.6061	0.1871
	rplB	0.4954	-0.0388
	rpsD	-0.4893	-0.3377
	rpsB	-0.5968	0.0882
	proS	-0.1880	-1.3832
	rplY	-0.4886	
	alaS	0.1329	-0.2954
	ileS	-0.2990	
	glyS	-0.0512	
	valS	-0.2595	-0.0876
	tsf	-0.6184	-0.4024
	infB		-0.1979
	rpsF		-0.4483
	rplP		0.2963
	rplX		-0.7096
	rplV		0.1803
	serS		-0.2051
**Amino acid metabolism**	***tnaA***	-0.6195	0.3862

	aspA	-0.4386	-0.1593
	carB	-0.2725	
	astC	0.2161	
	gabD	0.8170	
	dapD		-0.2999

Table S3: Statistical analysis of protein expression data for control and double knockout (DKO) strains: The proteins categorized under seven major functional categories (central carbon metabolism, translation, transcription, carbohydrate metabolism, energy metabolism, amino acid metabolism, protein sorting and folding) were selected from the list of top 100 abundant proteins for analysis. The statisticaly significant differences between log2 fold protein expression levels between control and DKO were analyzed by performing t-test for samples with unequal variances. P value ≤ 0.05 was considered significant.

(A) Statistical analysis for control. (FC represents 'fold change' between 4 h and 10 h post induction)

	**Functional categories**	**N1**	**Mean of Log2 FC (μ_1)**	**Variance (S_1^2)**	**St. Dev. (σ_1)**
1	Central carbon metabolism	24	-0.1258	0.2552	0.5051
2	Translation	20	-0.1832	0.1302	0.3608
3	Transcription	5	0.0887	0.3349	0.5787
4	Amino acid metabolism	5	-0.0595	0.2694	0.5190
5	Carbohydrate metabolism	4	-0.0854	0.1318	0.3631
6	Energy metabolism	4	-0.3048	0.0575	0.2398
7	Protein folding, sorting and degradation	4	0.0407	0.0600	0.2449

(B) Statistical analysis for DKO. (FC represents 'fold change' between 4 h and 10 h post induction)

	**Functional categories**	**N2**	**Mean of Log2 FC (μ_2)**	**Variance (S_2^2)**	**St. Dev. (σ_2)**
1	Central carbon metabolism	20	0.3277	0.1220	0.3493
2	Translation	23	-0.2189	0.1200	0.3464
3	Transcription	6	-0.0089	0.1540	0.3924
4	Amino acid metabolism	3	-0.0243	0.0876	0.2959
5	Carbohydrate metabolism	7	0.3202	0.6014	0.7755
6	Energy metabolism	6	0.3825	0.1358	0.3686
7	Protein folding, sorting and degradation	4	-0.0229	0.0409	0.2022

(C) t-test results (Control versus DKO)

	**Functional categories**	***t* value**	***p* value**
1	Central carbon metabolism	3.5055	0.0014
2	Translation	-0.3302	0.7488
3	Transcription	-0.3204	0.7829
4	Amino acid metabolism	0.1221	0.9201
5	Carbohydrate metabolism	1.1763	0.3166
6	Energy metabolism	3.5723	0.0152
7	Protein folding, sorting and degradation	-0.4004	0.7430

Figure S4: (A) Growth profile of *E. coli* BW25113 strain growing in TB media containing no glycerol, 0.2% and 0.4% (v/v) glycerol. (B) Glycerol consumption profiles of control and DKO strain growing in TB media containing 0.2% (v/v) glycerol.

(A)

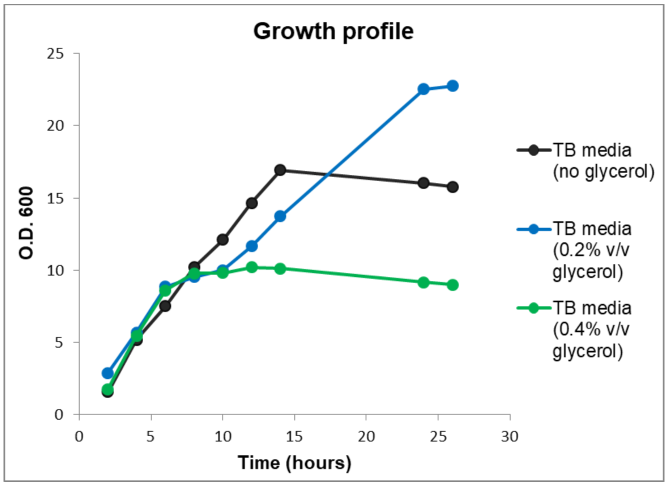

(B)

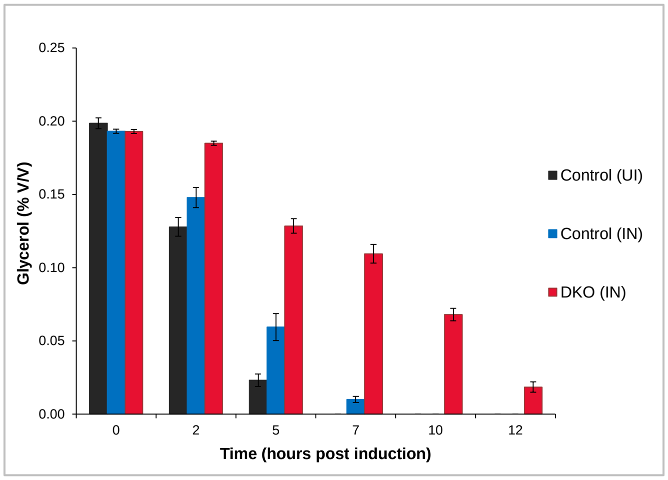

Figure S5: Confirmation of preserved functionality of *L-asparaginase* by estimating its specific activity.

Enzymatic activity measurements for *L-asparaginase*:

The enzymatic activity of *L-asparaginase* was quantified by measuring the amount of ammonia released during the reaction since it is directly proportional to the rate of hydrolysis of L-asparagine. A calibration curve of the amount of ammonia released vs OD_{436} was determined by Nessler's reagent using ammonium sulfate solution as standard. The enzymatic activity of the supernatant was quantified by measuring the maximum rate of substrate conversion, where one unit of *L-asparaginase* (U) is defined as the amount of enzyme required to convert 1 μmol of asparagine to 1 μmol ammonia per minute at 37°C and pH 8.6.

Calibration curve for calculating the amount of ammonia released:

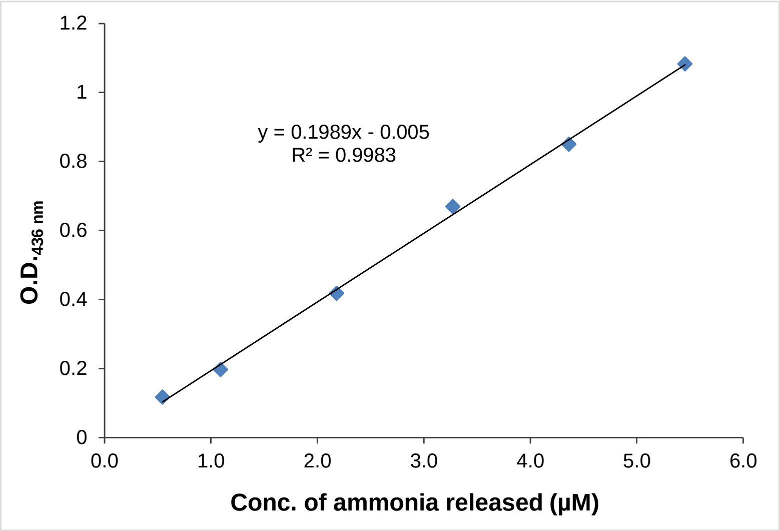

The enzymatic activity was calculated using the formula:

Units/ml Enzyme = (micromoles of NH_3 liberated) x (V1) **/** Assay time (min) x (V2) x (V3)

V1 = Total reaction volume (ml) before equilibration

V2 = Reaction volume (ml) used added to the well

V3 = Volume (ml) of enzyme solution/culture supernatant used

Calculations:

Strain	Time (hours PI)	Abs (436 nm)	Conc. of NH_3 liberated (in mM)	Enzyme activity (U/ml)
BW ΔelaAΔcysW L-asp	12	0.218	1.12	23.12
	24	0.334	1.70	35.15

BW ΔelaAΔcysW glpDK L-asp	12	0.292	1.49	30.80
	24	0.616	3.12	64.39

Gel densitometry studies:

Our preliminary studies showed that *L-asparaginase* starts getting secreted into the extracellular medium only after sufficient accumulation inside the cell. The expression of L-asparaginase in cytoplasmic and peri-plasmic fractions has been measured in some previous studies conducted by our lab (Khushoo *et al.,* 2004; Amardeep Khushoo, 2005). Since the extracellular secretion of this protein starts at 6 h post induction, we quantified the expression of *L-asparaginase* at 6, 12 and 24 h post induction (on 12% SDS gel) in DKO and DKO *glpDK* strains. Equal volumes of supernatant fractions were loaded on to SDS gel and the gel was stained with Coomassie blue. Gel densitometry studies performed by using ImageJ software (NIH, USA) confirmed approximately 2-fold increased expression levels for the double knockout strain containing *glpDK* genes.

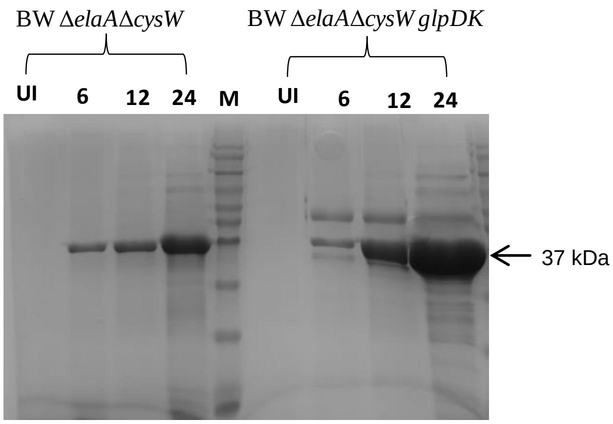

This 2-fold increase in *L-asparaginase* band intensity on SDS gel was also confirmed by loading different dilutions of the sample for the control and test strain in the gel followed by densitometric scanning. Since a similar increase in biological activity was observed for the test samples by the enzymatic assay it clearly demonstrates that the **specific enzymatic activity** of recombinant *L-asparaginase* remained the same in both samples. These results show that the higher level of expression in the double knock-out did not impact on the functionality of the expressed protein.

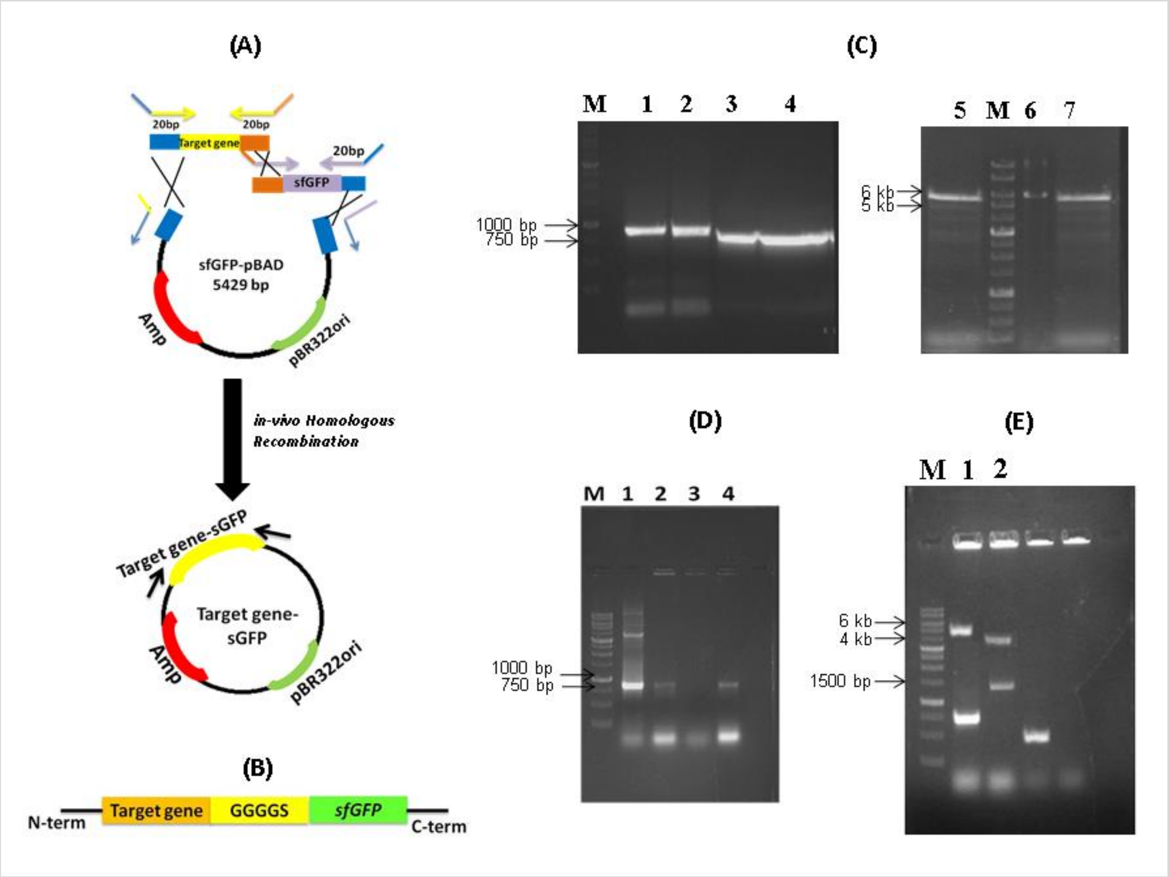

Figure S6: Cloning of Rubella E1 glycoprotein (target gene) and *sfGFP* into the *pBAD24* expression vector in *E. coli* DH5α strain using the principle of homologous recombination. The target gene was cloned between *NdeI* and *BglII*; and *sfGFP* gene was cloned between *BglII* and *HindIII* by introducing these restriction sites in primers designed for amplification. (A) Schematic showing the in vivo homologous recombination of target gene and *sfGFP* gene carrying a sequence for linker peptide (GGGGS) on their overlapping ends into *pBAD24* expression vector; (B) Schematic of functional gene cassette containing *sfGFP* as a reporter gene at its C-terminal end; (C) Agarose gel picture showing PCR amplification of target gene and *sfGFP* for cloning. Lane 1 & 2: PCR amplified target gene (789 bp); Lane 3 & 4: PCR amplified *sfGFP* gene (720 bp); Lane 5, 6 & 7: PCR amplified vector backbone (5.4 kb). (D) Screening of positive clones by forward and reverse primers (sequences shown in Table 1) for target gene. Lane M: 1 kb DNA ladder, Lane 1-4: Rubella E1 glycoprotein (789 bp). (E) Clone confirmation by double digestion. M: 1 kb DNA ladder; Lane 1: double digestion with *BglII* and *HindIII*; vector (5460 bp) and *sfGFP* gene fallout (720 bp); Lane 2: double digestion with *NdeI* and *HindIII*; vector (4595 bp) and Rubella E1-sfGFP construct fallout (1585 bp).

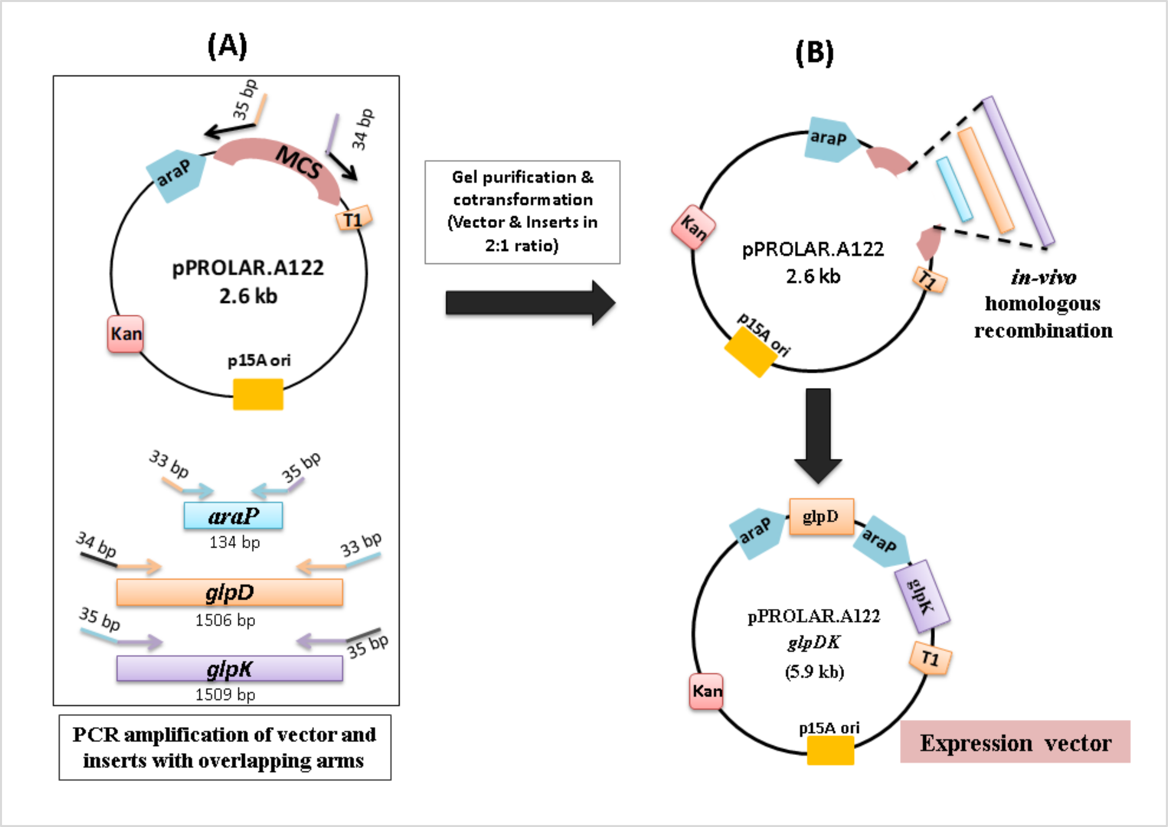

Figure S7: Pictorial representation of construction of expression vector *pPROLAR.A122glpDK* (5.9 kb). (A) Schematic showing PCR amplification of *araP* (ara promoter), *glpD* gene, *glpK* gene and vector backbone (*pPROLAR.A122*) using primers having 30-35 bp overhangs (containing sequences matching the end of next gene fragment to be cloned in respective direction); and (B) Schematic showing in vivo homologous recombination of *glpD* gene, ara promoter, *glpK* gene and vector backbone after co-transformation (vector to insert ratio of 2:1) into *E. coli* DH5α cells to form final expression vector.

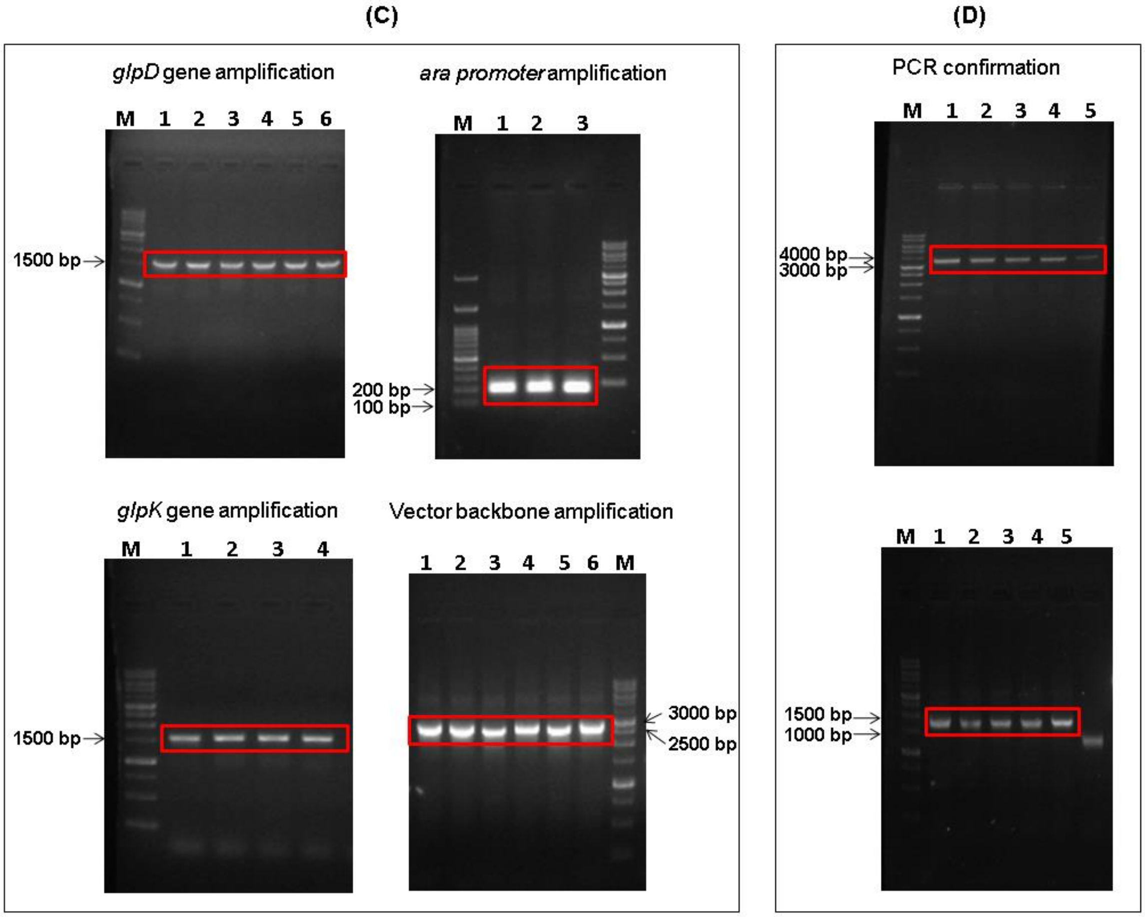

Figure S7: (C) Gel picture showing amplification of *glpD* gene (Lane M: 1 kb DNA ladder, Lane 1-6: *glpD* gene with overlaps (1573 bp)), Ara Promoter (Lane M: 1 kb DNA ladder, Lane 1-3: Ara promoters with overlaps (202 bp)), *glpK* gene (Lane M: 1 kb DNA ladder, Lane 1-6: *glpK* gene with overlaps (1577 bp)); and vector backbone (Lane M: 1 kb DNA ladder, Lane 1-6: vector backbone with overlaps (2.7 kb). (D) PCR confirmation of *glpD-araProm-glpK* insert (3.2 kb) by forward *glpD* and reverse *glpK* primers (shown in Table 1) and PCR confirmation of *araProm-glpK* insert (1.7 kb) by forward *araP* primer and reverse *glpK* primers.

Method S8: RNA-seq analysis procedure

A total of 3 µg of RNA was used for library construction. Paired end runs were performed on HiSeq 2500 platform (Illumina, Inc., USA) which provided the data in the form of 2 X 100 bp 30 million reads (3GB) per sample. The obtained Illumina HiSeq 2500 raw reads were trimmed for removing adapter sequences using Trimmomatic v.0.36 followed by the read quality assessment for each sample using FastQC v0.10.1. After quality assessment, reads were aligned to the reference genome of E. coli BW25113 which is available in the Ensemble database '*Escherichia coli* BW25113 ASM75055v1' in the Fasta-GFF3 format. The annotation file was obtained from ENSEMBL database in the GFF format and following steps were performed:

(a) Conversion of GFF annotation file to GTF format using "gffread" from Cufflinks suite.

(b) Reference indices were created using Bowtie2 tool and BAM files were generated.

(c) After performing alignment, mapped reads abundance was calculated using RSEM.

(d) Normalization was done by RSEM to rule out the effect of library size and reads length by estimating FPKM values for paired-end reads for each sample.

(e) Differential expression analysis was done using EdgeR software which is based on negative binomial distribution.

Pathway enrichment was done using the KEGG database. The total reads obtained for each sample lied in the range of 30-10 million reads with the mean read length of 100 bp. After trimming and filtering of reads, raw reads were mapped to the reference genome using Bowtie 2 tool with default settings. For both time-point samples of control and double-knock out strain as well as for respective single knockouts, the percentage of reads mapped was ranged from 95% to 65%. The raw reads and the processed data file have been deposited in the NCBI's GEO Database and are accessible through GEO series accession number GSE108442.

Differential expression analysis was performed using the edgeR software package. The expression of each gene for both strains were calculated and normalized in terms of FPKM values (fragments per kilo base of transcript per million mapped reads). We selected genes having $|\log2(X_{IN}/X_{UN})|>1$ i.e. a fold change of ≥2≤ and false-discovery-rate (FDR) corrected p value <0.05 for further analysis.

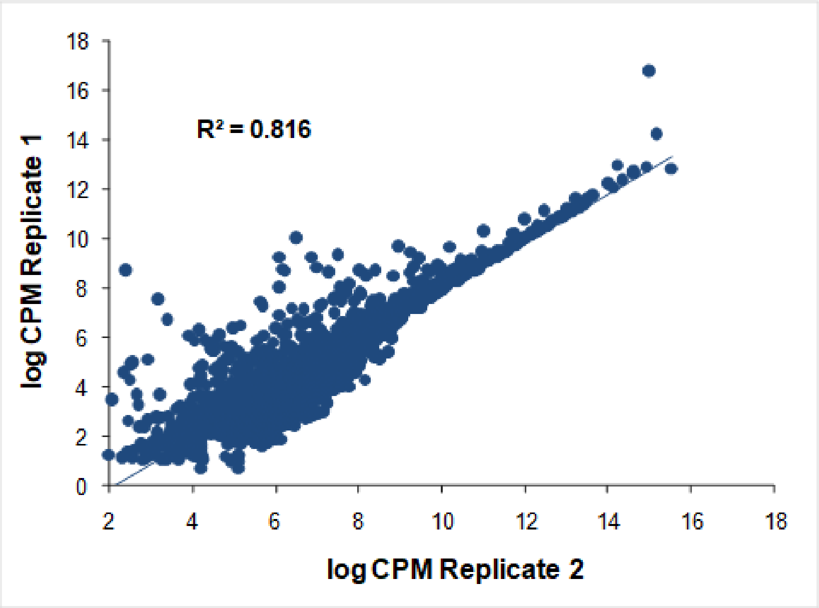

Figure S9: Correlation plot between log CPM values of two biological replicates of control strain (6 h post induction) expressing L-asparaginase. The correlation coefficient of 0.816 indicates a good degree of correlation between two biological replicates. CPM: counts per million

Method S10: qRT-PCR protocol

The genomic DNA depleted RNA samples were reverse transcribed using a random hexamer primer and Thermo Scientific RevertAid Reverse Transcriptase (RT). qRT-PCR was carried out in 7500 Fast Real-time PCR System (Applied Biosystems, Foster, USA). The reaction was performed using: 10 µl of Fast Universal SYBR Green (2x) Master Mix (Kapa Biosystems), 0.2 µl of 20 µM primer mix, 0.4 µl of ROX Reference Dye Low (50x), 1 µl of a diluted 1:5 cDNA template and water to complete a final volume of 20 µl. No template control (NTC) and positive control (genomic DNA) was also set up during the same reaction. All reactions were performed in technical triplicates. Cycling conditions were: initial denaturation at 95°C for 10 minutes, 40 cycles of 95°C denaturation for 15 s each, 60°C annealing for 1 min. After the final cycle, reaction specificity was verified by determining melting profiles over a temperature range of 65˚C to 95˚C in 0.2˚C increments. Resulted cycle threshold (C_t) values were average of biological duplicates for all genes. Reference gene (*rimL*, ribosomal-protein-L12-serine-acetyltransferase) C_t values were subtracted to achieve normalization of C_t values within the control and test sample genes (ΔC_t calculation). Fold changes were calculated using $2^{-\Delta\Delta CT}$ method and used for relative quantification log2 fold change.

Table S11: List of primers used for qRT-PCR.

S. No.	Name	Sequence (5' → 3')
1	*rimL RTF*	CGGTGCAGGGTAATGTGATG
2	*rimL RTR*	ATGAGATTCGTCCAGCCAGT
3	*rpoS RTF*	GCGACTCAGCTTTACCTTGG
4	*rpoS RTR*	CGTTCCGGGTCAAACTTCTC
5	*rpoD RTF*	CAGCGATACCTGGTTCAACG
6	*rpoD RTR*	CCATCTCTTTCTTCGCACGG
7	*glpK RTF*	CGGTCAGACTAACATTGGCG
8	*glpK RTR*	GCTTTCTCGCCAGTGTTCAT
9	*rpsF RTF*	CATGATCGAGCGCTACACTG
10	*rpsF RTR*	GGTAACAGCGTGCTTGGTAC
11	*gapA RTF*	TTTCCGTGCTGCTCAGAAAC
12	*gapA RTR*	GTCAACACCAACTTCGTCCC
13	*rmf RTF*	CTGGAACGGGCACATCAAC
14	*rmf RTR*	TTACTACCCTGTCCGCCATG
15	*ndh RTF*	AGCTACGGTTACAAAGGCCT
16	*ndh RTR*	CCATCTTTAGTGTGCAGGCC
17	*atpF RTF*	TGAGGCAGAACAGGAACGTA
18	*atpF RTF*	GTTAGCAGCTTCATCCACGG
19	*fis RTF*	CGTACTGACCGTTTCTACCG
20	*fis RTR*	CCACGGGTGTATTGCATCAC
23	*dps RTF*	GCGCTAACTTCATTGCCGTA
24	*dps RTR*	CCTGAACGTTGTGGATGTCC

(A)

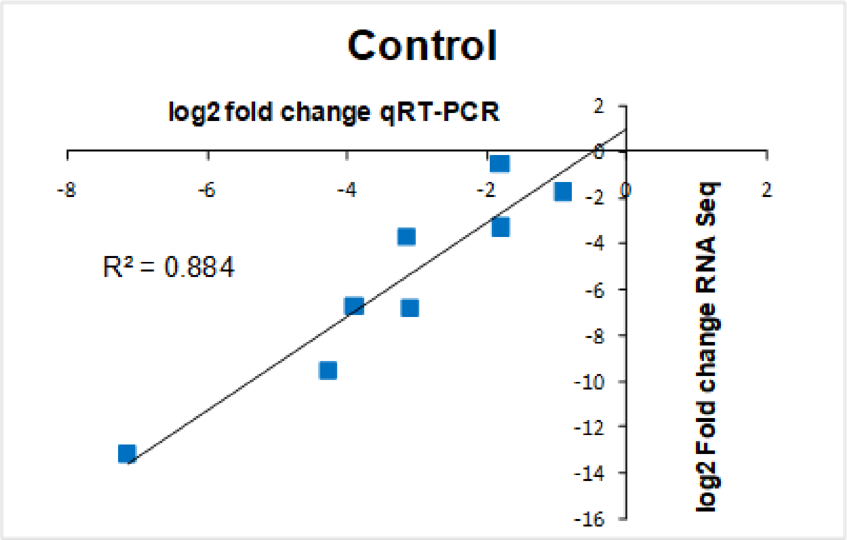

(B)

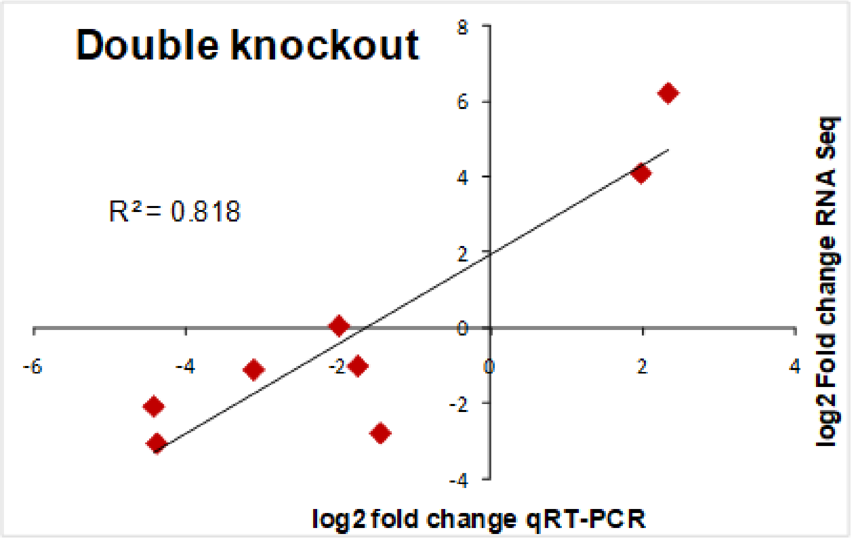

Figure S12: Correlation analysis of log2 fold change values obtained from RNA-Seq and qRT-PCR. RNA-Seq fold change refers to the ratios of log2 fold change values of 0 h and 6 h time points for selected transcripts of (A) control strain and (B) double knockout (DKO) strain, while qRT-PCR fold change is the relative quantity of 6 h time point normalized to expression level of 0 h time point.

Method S13: Label free LC MS/MS protein quantification

Mass spectrometry was performed on Orbitrap Velos mass spectrometer coupled with a Nano-LC 1000 system (Thermo Fisher Scientific). The peptides were separated on Acclaim PepMap 100 C18 precolumn (2 cm, 75 µm) connected to an Acclaim PepMap 100 C18 column (15 cm, 50 µm). Samples were run at flow rate of 300 nl/min using linear gradient of solvent B (95% acetonitrile containing 0.1% formic acid); 70 mins in 5-40% solvent B, 10 mins in 40-80% solvent B, 10 mins in 80% solvent B, 5 mins in 80-5% solvent B followed by 25 mins in 5% solvent B. Data were acquired under dynamic exclusion mode and the initial mass tolerance was kept 20 ppm, whereas the tolerance for the second mass spectrometer was 0.6 Da. MS/MS data was analyzed using Proteome Discoverer software (1.3.0.339 DBV version) using the SEQUEST algorithm. Spectra of peptides were queried against Uniprot knowledgebase for *E. coli* K-12 lineage containing decoy database using a target false discovery rate of 1% for strict and 5% for relaxed condition. Carbamidomethylation was kept as fixed modification and oxidation was kept as variable modifications. Trypsin was used as the enzyme of choice, and two missed cleavages were allowed. MaxQuant software was used for performing data normalization and analysis. Further analysis was done by sorting out data based on peptide score, protein coverage and peak area intensity values.

Table S14: Statistical analysis of gene expression data obtained from RNA seq analysis for control and double knockout (DKO) strains. The statistically significant differences between control and DKO were calculated using paired t-test performed on log2 fold gene expression levels for genes belonging to each functional category. Sample size (N) was kept same for both groups. GraphPad Prism Software (version 5) was used for performing statistical analysis. *p* values ≤0.05 are considered statistically significant. (The log2 fold change gene expression values for genes present in each functional category are given in Supplementary Table S1).

S. No.	Functional categories	N	*t* value	*df*	*p* value
1	Energy metabolism	25	5.086	39	< 0.0001
2	Transcription/translation	61	18.63	79	< 0.0001
3	Substrate uptake	7	2.648	11	0.0226
4	Central carbon metabolism	11	5.189	19	< 0.0001
5	Amino acid biosynthesis	26	4.566	34	< 0.0001
6	Cell motility	16	19.29	29	< 0.0001
7	Stress resistance	9	4.429	15	0.0005
8	Nutrient starvation	7	2.315	5	0.0538
